# An efficient exact algorithm for identifying hybrids using population genomic sequences

**DOI:** 10.1093/genetics/iyad011

**Published:** 2023-01-27

**Authors:** Sneha Chakraborty, Bruce Rannala

**Affiliations:** Graduate Group in Biostatistics, Department of Statistics, University of California at Davis, Davis, CA 95616, USA; Department of Evolution and Ecology, University of California at Davis, Davis, CA 95616, USA

**Keywords:** recombination, hybridization, whole-genome sequence data, conservation genetics, Bayesian inference

## Abstract

The identification of individuals that have a recent hybrid ancestry (between populations or species) has been a goal of naturalists for centuries. Since the 1960s, codominant genetic markers have been used with statistical and computational methods to identify F1 hybrids and backcrosses. Existing hybrid inference methods assume that alleles at different loci undergo independent assortment (are unlinked or in population linkage equilibrium). Genomic datasets include thousands of markers that are located on the same chromosome and are in population linkage disequilibrium which violate this assumption. Existing methods may therefore be viewed as composite likelihoods when applied to genomic datasets and their performance in identifying hybrid ancestry (which is a model-choice problem) is unknown. Here, we develop a new program Mongrail that implements a full-likelihood Bayesian hybrid inference method that explicitly models linkage and recombination, generating the posterior probability of different F1 or F2 hybrid, or backcross, genealogical classes. We use simulations to compare the statistical performance of Mongrail with that of an existing composite likelihood method (NewHybrids) and apply the method to analyze genome sequence data for hybridizing species of barred and spotted owls.

## Introduction

For hundreds of years, naturally occurring hybrids between species have been identified based on their morphology, often intermediate by comparison with the pure parental species. During the twentieth century, other diagnostics such as karyotypes, blood groups, and isozymes were increasingly used to identify hybrids. However, such diagnoses are inherently subjective and confounded by the presence of backcrosses. Codominant genetic markers potentially offer a more objective form of data and have been increasingly used to identify hybrid individuals in natural populations ever since the development of allozyme markers in the 1960s (Harris 1966; Lewontin and Hubby 1966). Genetic distance-based “hybrid indexes” were proposed in the 1970s, for example, to assign to individuals from known hybrid zones degrees of hybrid ancestry using allozyme allele frequencies of source populations (e.g. Hunt and Selander 1973). Applications of allozyme markers to identify population or species hybrids in animals and plants flourished during the 1980s (e.g.Grant *et al.* 1980; Joly and Adams 1983; Lamb and Avise 1986), and microsatellites (Roy *et al.* 1994) or AFLPs (Congiu *et al.* 2001) were commonly used during the 1990s and 2000s. The applications were often species conservation or, in the case of fish, stock management (Utter and Ryman 1993). During the 1980s, diagnostic criteria were proposed to identify hybrids based on explicit considerations of Mendelian inheritance. Some of these (e.g. Avise and Van Den Avyle 1984) rely on fixed allelic differences between pairs of populations (or species). If loci with fixed differences exist, F1 hybrids will be heterozygous for all such loci and (if markers are unlinked) the expected fraction of individuals that can be identified as F1 hybrids versus F2 backcrosses can be predicted as a function of the number of marker loci (Avise and Van Den Avyle 1984). Diagnostic statistics have also been developed to identify F1 or F2 hybrids and specific backcrosses (F1 × population 1, F1 × population 2) if one or more alleles are exclusive to each population but not necessarily fixed (Nason and Ellstrand 1993).

Fixed differences (or exclusive alleles) do not always exist between populations (or species) and (even if they do) it is impossible to be certain an allele is fixed (or exclusive) without exhaustive sampling. Another class of hybrid inference methods thus relax the requirement for fixed allelic differences, instead relying on differences in allele frequencies between populations (or species) and calculating probabilities of multilocus genotypes in F1 and F2 hybrids versus pure individuals (e.g. Campton and Utter 1985). Some methods for estimating individual proportions of admixture between populations (Rannala and Mountain 1997; Pritchard *et al.* 2000; Piry *et al.* 2004), or migrant ancestry (Wilson and Rannala 2003) also potentially identify F1 hybrids. In particular, Anderson and Thompson (2002) developed a powerful Bayesian method for distinguishing between hybrids and backcrosses based on multinomial sampling theory that has been widely used. In this paper, we focus on extending the hybrid inference method of Anderson and Thompson (2002) to accommodate genomic data using multilocus genotypes, or haplotypes, without a requirement for fixed or exclusive alleles.

### Assumptions of hybrid inference methods

Model-based methods for identifying hybrids assume random mating (Hardy–Weinberg equilibrium) within populations (species) and some form of independence among loci. The independence requirement (among loci) is usually stated as an assumption of either unlinked loci (e.g. Avise and Van Den Avyle 1984; Nason and Ellstrand 1993; Pritchard *et al.* 2000), or (linked or unlinked) loci that are in population linkage equilibrium (e.g. Campton and Utter 1985; Rannala and Mountain 1997; Anderson and Thompson 2002). The distinction can be important because markers on the same chromosome may be in linkage equilibrium, whereas none are unlinked.

The statistical assumption of independence among loci implies that the joint probability of alleles at multiple loci, calculated under a model with linkage, should be equal to the probability calculated as a product (across loci) of the marginal probabilities. This is generally stated without proof and thus axioms of the statistical methods are implicit. The more general requirement of statistical independence of alleles among loci seems to underlie both types of assumptions (unlinked loci or linkage equilibrium) in most methods. For large genomic datasets, many loci will be linked and in population linkage disequilibrium so that the assumptions of existing methods will be violated. Thus, alternative methods that relax these assumptions are desirable. A method that relaxes the independence assumption must formulate the problem in terms of linked markers on chromosomes and haplotype frequencies in populations, rather than multilocus genotypes and allele frequencies.

### Full versus composite likelihood

If a likelihood or Bayesian statistical method is applied that assumes unlinked loci, when the loci are actually linked, the method becomes a composite likelihood. Composite likelihoods can produce efficient point estimators, with estimates converging to the true parameter value with increased sample size (Varin *et al.* 2011). However, the problem of identifying hybrid class is a model choice problem not a point estimation problem—the performance of composite likelihood methods for model choice is poorly understood (Varin and Vidoni 2005). A full-likelihood method that explicitly models recombination, thus allowing linked loci, would eliminate the need for composite likelihood approximations and the potential problems of existing estimators. This paper aims to develop a full-likelihood method that explicitly models recombination during the formation of hybrids.

With the advent of genomic datasets of potentially millions of markers linked on chromosomes for which only a few recombination events are expected to occur per meiosis, the independence assumptions of composite likelihood methods will inevitably be violated. However, existing simulation studies examining the statistical performance of hybrid identification methods explicitly assume that markers are unlinked (Vähä and Primmer 2006). The effects of linkage on the performance of these composite likelihood methods are thus unknown. In particular, when thousands of markers are used so that the assumptions of the methods are strongly violated the effects of model violations could be extreme. Although the simulation method described in Wringe *et al.* (2019) includes a model of linkage, it has not yet been used to compare different hybrid inference methods. This paper develops a simulator that includes both linkage and recombination during the formation of population hybrids, allowing the statistical performance to be evaluated under a more realistic model for both existing (composite likelihood) hybrid inference methods and the new full-likelihood methods developed in this paper.

### Hybrid inference using genomic data with linked markers

There are 2 major factors accounting for the requirement, ubiquitous among existing hybrid inference methods, of independent assortment of alleles among loci: (1) the positions of first-generation genetic markers were usually unknown (a linkage or a physical map was not available) for most organisms; (2) explicitly modeling linkage and recombination is complex and computationally demanding. Advances in genome sequencing are rapidly altering this first factor, providing inexpensive physical maps of millions of SNP markers for virtually any species, and statistical methods and computer speed are rapidly converging to reduce or eliminate the second limiting factor (computational complexity).

In this paper, we present a full-likelihood Bayesian method for identifying hybrids and backcrosses using biallelic SNP loci available from population genomic samples. The method explicitly models recombination by using a physical map of the markers. To maximize the efficiency of the method we implement many of the computations as low-level bit operations in the C programming environment. To examine the effects of linkage on composite likelihood methods, we conducted a simulation study of the performance of the Anderson and Thompson (2002) composite likelihood method, when used with linked markers, by comparison with our full-likelihood method which incorporates linkage. As an application of our method, we analyze a real dataset consisting of spotted owls (SO), barred owls (BO), and their hybrids (Hanna *et al.* 2018; Fujito *et al.* 2021). This dataset includes an improved spotted owl genome assembly and 51 high coverage whole-genome sequences (Fujito *et al.* 2021).

## Theory

Most methods using genetic markers to identify population hybrids of a diploid species have assumed that genotypes at different loci undergo independent assortment (e.g. are unlinked or in linkage equilibrium). In particular, the widely used method of Anderson and Thompson (2002) incorporates this assumption—when markers are actually linked their method becomes a composite likelihood approximation. Here, we extend the Anderson and Thompson (2002) model to allow genomic sequence data comprised of linked SNPs to be jointly analyzed using an exact full-likelihood approach. We consider 2 sympatric diploid populations, labeled A and B, and assume they were initially isolated but have been interbreeding for the last *n* generations. We follow Anderson and Thompson (2002) who considered all the combinations of hybrid ancestries that can result with *n* generations of interbreeding between 2 populations but consider explicit results only for the case of a recent population hybridization event (*n* ≤ 2 generations). Here, we consider the diploid genome sequence for a single individual and how this may be used to infer the hybrid status of the individual. In the absence of close relatedness between sampled individuals the hybrid status of multiple individuals may be inferred independently.

### Genealogical class

**Fig. 1.**
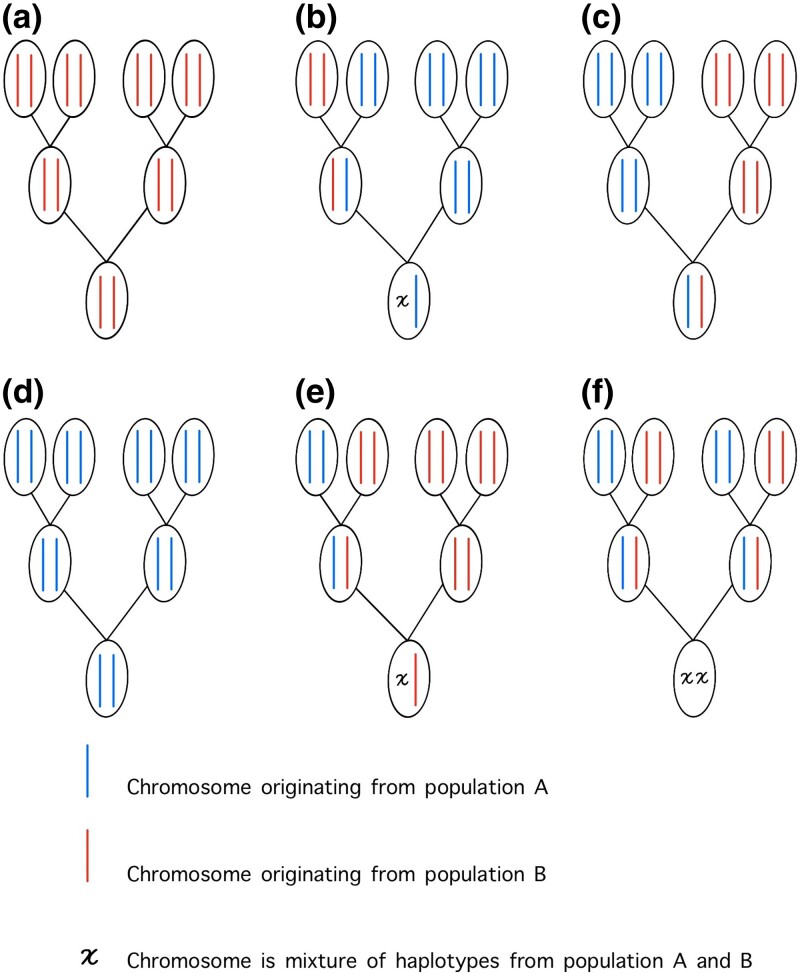
Pedigrees of relationships among founders for *n* = 2 generations. Circles represent diploid individuals and the pair of lines within each individual represent the 2 chromosomes; the blue line denotes the chromosome originating from population A and the red line represents the chromosome from population B. Founders are individuals at the top of the pedigree. An individual’s genealogical class (a–f), is defined by the population origins among founders.

A noninbred pedigree of *n* generations describing the ancestors of a single individual includes 2^*n*^ founders, so in our 2 generation case, there are 2^2^ = 4 founders. We consider the founders to be purebred. We can identify 6 distinct classes of such 2-generational pedigrees by considering the number and arrangement of founders originating from a specified population (population A, for example). Following Nason and Ellstrand (1993), we refer to these as genealogical classes (see Fig. 1), where genealogical classes **a** and **d** are purebreds; **b** and **e** are backcrosses; **c** is a F1 hybrid and **f** is a F2 hybrid. We use the term genealogical class and model interchangeably. Our approach differs from Nason and Ellstrand (1993) in that we consider diplotypes rather than marker genotypes. The diplotype is the pair of haplotypes on homologous chromosomes.

If the map distances between markers and the population haplotype frequencies are known, then under a random mating process (within populations) the probability of the observed marker data (likelihood) is completely specified for any given genealogical class. The focus of this paper will be on developing and implementing an efficient algorithm for calculating this likelihood, thus allowing inference of the genealogical class of an individual using linked SNP marker data.

### Model and likelihood

Here, we describe the likelihood calculation for a set of multiple biallelic SNP markers located on the 2 copies (maternal and paternal) of a single chromosome from an individual of a diploid sexual species that undergoes recombination. Because different chromosomes segregate independently during meiosis, the likelihood for markers on multiple chromosomes is simply a product across the likelihoods for the individual chromosomes. Following Anderson and Thompson (2002), the objective of the inference will be to infer the hybridization history of an individual (genealogical class) which is essentially a model choice problem. We will use posterior model probabilities to evaluate the support for different genealogical classes. Here, we present the data, model parameters, and formulas needed for calculating the likelihood under each of the 6 possible genealogical classes (see Fig. 1) for 2 populations.

#### Data and parameters

Consider a sample of *K* chromosomes from a diploid individual. Chromosome *i* contains *L*_*i*_ loci with phased biallelic single-nucleotide polymorphisms (SNPs). We represent the maternally (*M*) and paternally (*P*) inherited copies of the chromosomes as matrices,


$$\begin{array}{rl} x^{M} & =\{x_{ij}^{M}\},\\ x^{P} & =\{x_{ij}^{P}\}, \end{array}$$

where $x_{ij}^{M}\in \{0,1\}$ is the allele (coded as 0,1) present at the *j*th SNP locus on the maternally inherited copy of chromosome *i*, etc. The complete data for an individual are then **x** = {**x** ^*M*^, **x** ^*P*^}. We define the physical distances between markers on chromosomes as


$$d=\{d_{ij}\},$$

where *d*_*ij*_ is the distance on chromosome *i* from marker *j* − 1 to *j* and *d*_*i*1_ is the distance from the 5’ end of chromosome *i* to marker 1.

We define the recombination rates on the intervals between markers as


$$r=\{r_{ij}\},$$

where *r*_*ij*_ is the recombination rate on chromosome *i* for the interval between markers *j* − 1 and *j* in units of centiMorgans (cM) per unit of physical distance. For example, if physical distance is measured in megabases (Mb), the units would be cM/Mb. Recall that 1cM=1% recombination per meiosis. The map distance for the interval [*j* − 1, *j*] of chromosome *i* is defined by the product *d*_*ij*_ × *r*_*ij*_ and is measured in units of cM (percent recombination per meiosis).

We consider a model with hybridization between 2 populations A and B. The ancestry matrix for each chromosome specifies the population origin of each marker locus,


$$\begin{array}{rl} z^{M} & =\{z_{ij}^{M}\},\\ z^{P} & =\{z_{ij}^{P}\}, \end{array}$$

where populations A and B are denoted by 0 and 1, respectively, and $z_{ij}^{M}=0$ specifies that marker *j* of the maternally inherited copy of chromosome *i* originates from a founder chromosome that was in population A, and so on. The complete ancestry matrix for an individual is then **z** = {**z** ^*M*^, **z** ^*P*^}.

Population haplotype frequencies are also needed to calculate likelihoods. We assume that the population is randomly mating so that diplotype probabilities can be calculated directly from haplotype frequencies for nonhybrid segments of chromosomes. We define *f* ^*A*^(*x*_*i*_) to be the frequency of haplotype *x*_*i*_ on chromosome *i* in population A and *f* ^*B*^(*x*_*i*_) its frequency in population B. In this paper, we treat population haplotype frequencies as known when calculating likelihoods. In empirical analyses, haplotype frequencies are estimated using individuals who are unlikely to be hybrids (for example, individuals sampled outside of a hybrid zone). For the individual being tested for potential hybrid status, we integrate over the unknown haplotype phase taking account of uncertainty (see section Inference for unphased individuals).

#### Population ancestry states of markers

**Fig. 2.**
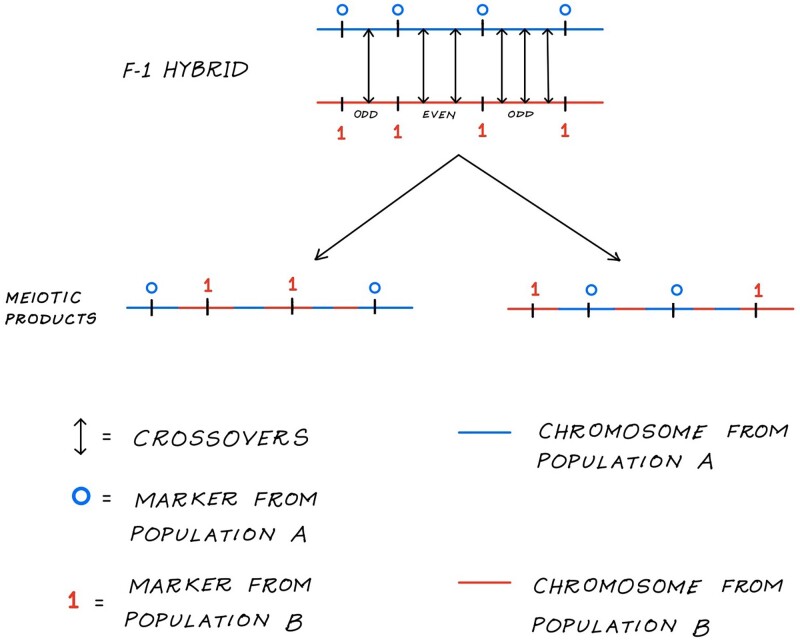
Population origin of *L* = 4 markers as a result of recombination (or, crossovers) between 2 pure chromosomes (a F1 hybrid): 1 from population A (blue) and other from population B (red). The 2 possible gametes (recombinant haplotype) produced at the end of meiosis is shown with the population origin labeled on the markers. An odd number of recombinations between 2 markers changes the population origin of the marker to the right of an interval whereas an even number of recombinations results in no change in the population state of the markers.

For the case of 2 populations and 2 generations of hybridization, the only nontrivial chromosome ancestry probability to calculate is that for a chromosome that is a recombinant between 2 pure chromosomes, 1 from population A and the other from population B. We consider autosomes so that it does not matter which specific chromosome is maternally, or paternally, inherited. We assume no interference (independence of recombination events on different intervals) allowing the transitions from 1 population ancestry state to another along the chromosome from left to right (see Fig. 2) to be calculated as independent conditional probabilities. Here, we present the probability calculation for chromosome *i* with *L*_*i*_ linked loci. We omit the subscript *i* from the population ancestry vector *z* for simplicity.

The population origin of a SNP locus to the right of an interval changes whenever there is an odd number of recombinations (1,3,5, etc.) on the interval. We denote a distinct population state (or, ancestry state) as


$$z=\{z_{\;j}\};\;j=1,\ldots ,L_{i},$$

where *z*_*j*_ ∈ {0, 1} is the population origin of the marker *j* (where 0 represents population A and 1 population B).

There are $2^{L_{i}}$ possible distinct population states for *L*_*i*_ loci, for example:


$$\underset{L_{i}}{\underbrace{0\cdots 0}},\underset{L_{i}}{\underbrace{1\cdots 1}},\underset{L_{i}-1}{\underbrace{0\cdots 0}}\underset{1}{\underbrace{1}},\underset{L_{i}-2}{\underbrace{0\cdots 0}}\underset{2}{\underbrace{11}},\ldots$$

For simplicity, here we treat the rate of recombination as uniform (*r*_*ij*_ = *r*) on chromosome *i*, although the implementation allows recombination rates to vary as specified by the user. Given the assumption of no interference, recombinations occur as a Poisson process along the chromosome, such that the number of recombinations in an interval of length *d*_*ij*_ is Poisson distributed with a mean of *rd*_*ij*_. Accordingly, the probability that an even number of recombinations occur on an interval of length *d*_*ij*_ is


$$\sum_{n=1}^{\infty }\frac{e^{-rd_{ij}}(rd_{ij}{)}^{2n}}{[2n]!}=e^{-rd_{ij}}(\text{cosh}[rd_{ij}]-1).$$

Thus the probability that there is a change of population on the interval between the (*j* − 1)th marker and *j*th marker is


$$P(d_{ij},r)=1-(e^{-rd_{ij}}\{\text{cosh}[rd_{ij}]-1\}+e^{-rd_{ij}}),$$

where the last term in parentheses on the right is the probability of no recombinations (which also results in no change of population ancestry). Using this result, we can calculate the probability of a particular ancestry state **z** for *L*_*i*_ SNP loci,


$$\begin{array}{rl} Q(z\;|\;d_{i},r) & =\left\{\frac{1}{2}\times {P(d_{i1},r)}^{z_{1}}\times {\left[1-P(d_{i1},r)\right]}^{1-z_{1}}\times P^{*}\right\}\\ & \;+\left\{\frac{1}{2}\times {P(d_{i1},r)}^{\left|z_{1}-1\right|}\times {\left[1-P(d_{i1},r)\right]}^{1-|z_{1}-1|}\times P^{*}\right\}, \end{array}$$

where


$$P^{*}=\prod_{k=2}^{L_{i}}\left\{{P(d_{ik},r)}^{\left|z_{k}-z_{k-1}\right|}\times {\left[1-P(d_{ik},r)\right]}^{1-|z_{k}-z_{k-1}|}\right\}.$$

Here, we explain the derivation of *Q*(**z** | *d*_*i*_, *r*) for a particular ancestry state **z**. A chromosome can either be sampled from population A (with probability 1/2) or from population B (with probability 1/2). This explains the summation of the 2 (mutually exclusive and exhaustive events) represented by the terms in curly braces. Considering the first term, given that the chromosome was sampled from population A, if *z*_1_ = 0 no change of population state occurred on interval *d*_*i*1_. The probability of no change is [1 − *P*(*d*_*i*1_, *r*)]. Otherwise, if *z*_1_ = 1 the population state changed on interval *d*_*i*1_. The probability a change occurs is *P*(*d*_*i*1_, *r*). The derivation for a chromosome sampled from population B (the second term) is similar. For the remaining loci (*z*_*k*_; *k* > 1), the probability is *P*(*d*_*ik*_, *r*) if the ancestry state *z*_*k*_ changed from *z*_*k*−1_, otherwise the probability is [1 − *P*(*d*_*ik*_, *r*)] if there was no change (*z*_*k*_ = *z*_*k*−1_; *k* ≠ 1), and these probabilities are combined into the term P*.

### Allelic state of markers

We now consider the probabilities of the SNP alleles observed conditional on the ancestry states of the markers. Let *x*_*i*_ be the SNP haplotype for a (maternally or paternally inherited) chromosome *i* and let **z** be the population ancestry vector (again dropping the chromosome *i* subscript for simplicity). The probability of the SNP alleles on the chromosome for the 2 trivial cases (either the entire chromosome comes from population A or from population B) are given by


$$f(x_{i}\;|\;z)=\left\{ \begin{array}{cc} f^{A}(x_{i}), & \text{if }z_{j}=0\;\forall \;j=1,\ldots ,L_{i}\\ f^{B}(x_{i}), & \text{if }z_{j}=1\;\forall \;j=1,\ldots ,L_{i}. \end{array}\right.$$

Now consider the nontrivial case (i.e. the chromosome is a recombinant between 2 pure chromosomes, 1 from population A and the other from B). First, we define the marginal frequency of alleles on sub haplotypes {*x*_*iu*_, *x*_*i*(*u*+1)_, *x*_*i*(*u*+2)_, …, *x*_*iv*_} from the *u*th marker to the *v*th marker where 1 ≤ *u* < *v* ≤ *L*_*i*_ in population A (or, B) be *f* ^*A*^(*x*_*i*[*u*,*v*]_) or, *f* ^*B*^(*x*_*i*[*u*,*v*]_), respectively. Then, for any ancestry state **z** we consider each segment of consecutive 0’s or 1’s, counting the number of segments and the lengths of each segment (in units of number of markers rather than physical distance). Consider the case of *c* segments with lengths *j*_1_, *j*_2_, …, *j*_*c*_. The calculation differs for even versus odd numbers of segments.

*Case I:* Even number of segments *c* = 2*n* and *z*_1_ = 0 (i.e. the starting state is population A),


$$\begin{array}{rl} \;f(x_{i}\;|\;z) & =f^{A}(x_{i[1,j_{1}]})\\ & \;\times f^{B}(x_{i[j_{1}+1,j_{1}+j_{2}]})\\ & \;\times f^{A}(x_{i[j_{1}+j_{2}+1,j_{1}+j_{2}+j_{3}]})\\ & \;\vdots \\ & \;\times f^{B}(x_{i[j_{1}+j_{2}+\cdots +j_{c-1}+1,j_{1}+j_{2}+\cdots +j_{c}]}). \end{array}$$

The terms are thus an alternating sequence of marginal frequencies from populations A and B, and there are *n* marginal frequency terms from each. If *z*_1_ = 1, the first term instead starts with population B and the last term is from population A.

*Case II:* Odd number of segments *c* = 2*n* + 1 and *z*_1_ = 0 (i.e. the starting state is population A),


$$\begin{array}{rl} \;f(x_{i}\;|\;z) & =f^{A}(x_{i[1,j_{1}]})\\ & \;\times f^{B}(x_{i[j_{1}+1,j_{1}+j_{2}]})\\ & \;\times f^{A}(x_{i[j_{1}+j_{2}+1,j_{1}+j_{2}+j_{3}]})\\ & \;\vdots \\ & \;\times f^{B}(x_{i[j_{1}+j_{2}+\cdots +j_{c-2}+1,j_{1}+j_{2}+\cdots +j_{c-1}]})\\ & \;\times f^{A}(x_{i[j_{1}+j_{2}+\cdots +j_{c-1}+1,j_{1}+j_{2}+\cdots +j_{c}]}) \end{array}$$

In this case (*z*_1_ = 0), there are (*n* + 1) marginal frequency terms from population A and *n* from B. If *z*_1_ = 1, the first and last terms instead come from population B so that (*n* + 1) marginal frequency terms are from population B and *n* from A.

### Likelihood

For the maternally inherited chromosome *i*, the probability of a *L*_*i*_-loci hybrid SNP haplotype (i.e. a recombinant SNP haplotype), $x_{i}^{M}$, unconditioned on the ancestry state of the markers is given by


$$\begin{array}{rl} L(x_{i}^{M}\;|\;d_{i},r) & =\sum_{z}L(x_{i}^{M},z\;|\;d_{i},r)\\ & =\sum_{z}f(x_{i}^{M}\;|\;z).Q(z\;|\;d_{i},r), \end{array}$$

where the sum is over the set of all possible ancestry states at *L*_*i*_ markers (there are $2^{L_{i}}$ distinct combinations). Because the number of distinct combinations grows with *L*_*i*_, it is currently computationally practical to analyze 15 or fewer loci per chromosome.

### Likelihoods for genealogical classes

Here, we present formulas for calculating the likelihood of a diplotype, **x** = {**x** ^*M*^, **x** ^*P*^} for an individual under each of the 6 possible genealogical classes (see Fig. 1). We use the term genealogical class and model interchangeably. We introduce the variable *G* : *G* = *g* denotes that the individual belongs to the genealogical class *g*. We define the indicator function,


$$I=\left\{ \begin{array}{cc} 1 & \text{if }x_{i}^{M}\neq x_{i}^{P}\\ 0 & \text{if }x_{i}^{M}=x_{i}^{P}. \end{array}\right.$$

*Model (a)*: Both chromosomes ($x_{i}^{M}$ and $x_{i}^{P}$) come from Population B (i.e. $z_{j}=1\;\forall \;j=1,\ldots ,L_{i}$)


$$L(x^{M},x^{P}\;|\;G=1,d,r)=\prod_{i=1}^{K}\{2^{I}.f^{B}(x_{i}^{M}).f^{B}(x_{i}^{P})\},$$

*Model (b)*: One chromosome comes from Population A, and the other chromosome is a recombinant.


L(xM,xP|G=2,d,r)=∏i=1K([fA(xiM){∑zf(xiP|z).Q(z|di,r)}[fA(xiM){∑zf(xiP|z).Q(z|di,r)}]1−I+fA(xiP){∑zf(xiM|z).Q(z|di,r)}]I×[fA(xiM){∑zf(xiP|z).Q(z|di,r)}]1−I),

*Model (c)*: One chromosome comes from Population A and the other from Population B


$$\begin{array}{rl} & L(x^{M},x^{P}\;|\;G=3,d,r)\\ & \;=\prod_{i=1}^{K}[{\left\{\;f^{A}(x_{i}^{M}).f^{B}(x_{i}^{P})+f^{A}(x_{i}^{P}).f^{B}(x_{i}^{M})\right\}}^{I}\\ & \;\times {\left\{\;f^{A}(x_{i}^{M}).f^{B}(x_{i}^{P})\right\}}^{1-I}], \end{array}$$

*Model (d)*: Both chromosomes ($x_{i}^{M}$ and $x_{i}^{P}$) come from Population A (i.e. $z_{j}=0\;\forall \;j=1,\ldots ,L_{i}$)


$$L(x^{M},x^{P}\;|\;G=4,d,r)=\prod_{i=1}^{K}\{2^{I}.f^{A}(x_{i}^{M}).f^{A}(x_{i}^{P})\},$$

*Model (e)*: One chromosome comes from Population B and the other chromosome is a recombinant.


L(xM,xP|G=5,d,r)=∏i=1K([fB(xiM){∑zf(xiP|z).Q(z|di,r)}[fB(xiM){∑zf(xiP|z).Q(z|di,r)}]1−I+fB(xiP){∑zf(xiM|z).Q(z|di,r)}]I×[fB(xiM){∑zf(xiP|z).Q(z|di,r)}]1−I),

*Model (f)*: Both chromosomes are recombinants.


$$\begin{array}{rl} & L(x^{M},x^{P}\;|\;G=6,d,r)\\ & \;=\prod_{i=1}^{K}\left[2^{I}.\left\{\sum_{z}f(x_{i}^{M}\;|\;z).Q(z\;|\;d_{i},r)\right\}.\left\{\sum_{z}f(x_{i}^{P}\;|\;z).Q(z\;|\;d_{i},r)\right\}\right] \end{array}$$

Likelihoods for 3 genealogical classes (a, d, and c) shown in Fig. 1 are trivial to calculate because recombination has no effect on the population origins of linked sites. For models (a) and (d), the sampled individual’s chromosomes are either entirely A or entirely B. For model (c), the individual is a first-generation hybrid so that one chromosome is entirely A and the other entirely B. The probability of the data for these genealogical classes can therefore be calculated directly by assuming random mating and applying the usual Hardy Weinberg formulas using the population haplotype frequencies in place of allele frequencies. Models (b), (e), and (f) all require calculation of the probabilities of one or more recombinant haplotypes (indicated by an *x* in Fig. 1).

### Bayesian inference

A Bayesian approach to hybrid inference requires a prior distribution for the genealogical classes. An individual belongs to genealogical class *g* with prior probability *π*_*g*_ with *g* = 1, 2, …6 and $\sum_{g=1}^{6}\pi_{g}=1$. The posterior probability an individual belongs to the *g*th genealogical class is


$$P(G=g\;|\;x^{M},x^{P},d,r)=\frac{\pi_{g}\times P(x^{M},x^{P}\;|\;G=g,d,r)}{\sum_{i=1}^{6}\pi_{i}\times P(x^{M},x^{P}\;|\;G=i,d,r)}.$$

In general, a prior distribution on genealogical classes could incorporate factors such as differential fitnesses among classes, varying frequencies of mating encounters between individuals of different populations (or species), and so on. Lacking specific information on hybridization rates, one can use a discrete uniform prior $\pi_{g}=1/6,\;\forall \;g=1,\ldots ,6$ which reduces the posterior likelihood to


$$P(G=g\;|\;x^{M},x^{P},d,r)=\frac{P(x^{M},x^{P}\;|\;G=g,d,r)}{\sum_{i=1}^{6}P(x^{M},x^{P}\;|\;G=i,d,r)}.$$

### Estimation of population haplotype frequencies

The likelihood theory above treats population haplotype frequencies as known fixed parameters. For most empirical datasets, population haplotype frequencies are unknown; we thus estimate them using posterior probability densities. To estimate frequencies “purebred” individuals (those least likely to be hybrids; possibly sampled outside a hybrid zone) are identified from each of the 2 populations and analyzed separately. Let **f^i^** = {*f* ^*i*^(*h*)} be a vector of the haplotype frequencies in population *i*, where *f* ^*i*^(*h*) is the frequency of the *h*th distinct haplotype in *i* and *i* ∈ {*A*, *B*}. Let *N*_*i*_ be the number of diploid individuals sampled from population *i*. It is assumed that *H* distinct haplotypes exist, each occurring in both populations. The set of distinct haplotypes compatible with genotypes observed in all sampled individuals (from both populations) provides an estimate of *H*. With no prior information, we assign equal prior probability density to the haplotype frequencies in each population (A and B). Results are formulated for population A (B is equivalent). The prior probability density of haplotype frequencies (i.e. before sampling) is


$$\text{Pr}(f^{A})=\prod_{h=1}^{H}\frac{\{f^{A}(h){\}}^{(1/H)-1}}{\Gamma \left(1/H\right)}.$$

Thus, **f^A^** ∼ Dirichlet(1/*H*). Let the vector **n**_*A*_ = {*n*_1*A*_, …, *n*_*HA*_}, where *n*_*hA*_ is the observed number of copies of the *h*th distinct haplotype in a sample from population A. The probability density of **n**_*A*_ conditioned on the haplotype frequencies follows a Multinomial distribution given by


Pr(nA|fA)=(∑h=1HnhAn1A,…,nHA)∏h=1H{fA(h)}nhA,

where


$$\sum_{h=1}^{H}n_{hA}=2N_{A}$$

is the total number of haplotypes observed in population A. The posterior density of the haplotype frequencies, conditioned on the haplotypes observed in a sample from population A, follows a Dirichlet distribution, since the Dirichlet is a conjugate prior to a Multinomial distribution. The posterior probability density of haplotype frequencies is


$$\text{Pr}(f^{A}\;|\;n_{A})=\Gamma \left(\theta_{A}\right)\prod_{h=1}^{H}\frac{\{f^{A}(h){\}}^{\theta_{A}a_{hA}-1}}{\Gamma \left(\theta_{A}a_{hA}\right)},$$

where


$$\theta_{A}=\left(1+\sum_{h=1}^{H}n_{hA}\right)=(1+2N_{A})$$

and


$$a_{hA}=\frac{n_{hA}+1/H}{1+\sum_{h=1}^{H}n_{hA}}=\frac{n_{hA}+1/H}{1+2N_{A}}.$$

Note that $\sum_{h=1}^{H}a_{hA}=1$. The posterior mean is used to estimate **f^A^** and is given by


(1) $$E(f^{A}\;|\;n_{A})=\frac{\theta_{A}a_{hA}}{\sum_{h=1}^{H}(\theta_{A}a_{hA})}=a_{hA}.$$

For simplicity, we ignore uncertainties of haplotype population frequencies when inferring hybrid classes, using the posterior mean as a proxy for the true frequency. Uncertainties could be accounted for by instead integrating over the posterior density rather than using the posterior mean.

### Inference for unphased individuals

The above calculations require phased haplotypes for all individuals. Although improved genomic sequencing technologies provide more experimental information about phase than in the past, complete phase information is not always available. For the “pure” individuals, one can obtain phase by applying existing population-based haplotype phasing methods (Stephens *et al.* 2001; Browning and Browning 2007; Browning *et al.* 2021) to each population separately. However, with unphased putative hybrid individuals using either pure population for phasing would be incorrect, and thus could potentially lead to biased inferences, unless the individual turns out to be a nonhybrid. Therefore, instead of trying to estimate the phase of a hybrid individual, we opted to integrate over all possible haplotype phase states.

Let **X** = {*X*_*ij*_} be the matrix of genotype data of a hybrid individual, where *X*_*ij*_ is the genotype at the *j*th SNP locus for chromosome *i*. Given the unphased multilocus genotype data *X*_*i* ***·***_ for chromosome *i*, let *C*_*i*_ denote its set of compatible diplotypes. Thus for chromosome *i*, given the *g*th genealogical class, the likelihood of the unphased hybrid individual is


(2) $$P(X_{i\cdot }\;|\;G=g,d,r)=\sum_{\{x_{i}^{M},x_{i}^{P}\}\in C_{i}}P(x_{i}^{M},x_{i}^{P}\;|\;G=g,d,r).$$

Here, we are summing over all compatible diplotypes to obtain the marginal likelihood, taking into account the uncertainty in phasing. For a sample of *K* chromosomes, the likelihood of the unphased hybrid is


(3) $$\prod_{i=1}^{K}P(X_{i\cdot }\;|\;G=g,d,r).$$

The chromosome probabilities are multiplied because the different chromosomes undergo independent assortment during meiosis. These equations were implemented in a new inference program named Mongrail.

## Simulation study

Two different simulation study designs were used to evaluate the statistical performance of our new inference method Mongrail versus NewHybrids. The first design involved generating a diverse set of haplotype frequency distributions, ensured by using either a symmetrical or nonsymmetrical Dirichlet distribution. This allowed a comprehensive comparison of performance over a broad range of conditions. The second design used a structured coalescent model with recombination, with haplotypes generated under a neutral Wright–Fisher model from each of 2 populations (A and B) connected by migration, allowing the statistical performance of the inference method to be evaluated under biologically realistic conditions.

### Comprehensive simulation

We simulated diplotypes for individuals that were uniformly assigned to 1 of the 6 genealogical classes. The chromosomes of parents were randomly assigned haplotypes according to the population haplotype frequencies in populations A and B, which must first be specified. We first describe the procedure used to simulate haplotype marker configurations and their corresponding frequencies (for 2 populations A and B) for each chromosome. We use simple procedures designed to mimic population genetic processes rather than explicitly simulating a population genetic model as it allowed more direct control over levels of variation in the populations. For simplicity, in all simulations, we fixed the length of each chromosome to be 240 Mb and the recombination rate to be 1.2 cM/Mb, respectively. The simulation experiment used a factorial design allowing the performance of the methods to be assessed for many combinations of parameters. The parameters (factors) and their values were as follows:

1. Number of chromosomes: *K* = 1, 2, 5, 10, 20
2. Number of loci per chromosome: *L* = 1, 5, 10
3. Expected recombination frequency (in cM): *R* = 1, 25, 50 between the first and the last locus
4. Number of distinct haplotype sequences per chromosome for each population: *h* = 5, 10, 15
5. Allelic configurations of haplotypes, generated by simulating the switches between allele states (see description below). Switch rates used: *c* = 0.1, 1, *L*/2
6. Haplotype frequencies, following a Dirichlet distribution (symmetrical with parameters *α* = 1, 5, or nonsymmetrical with parameters *w* = 5, 20) (see description below)

To simulate haplotype configurations, we mimic recombination by using a “switching process” (that flips the adjacent marker state) operating along the chromosome. The switch rate on a particular interval is *p* = *c*/*L*, where *p* is the probability of a switch from 0 to 1 (or 1 to 0). To simulate haplotype frequencies, we used either a symmetrical or nonsymmetrical Dirichlet distribution with parameters *α*_1_, *α*_2_, …, *α*_*h*_. For the symmetric Dirichlet distribution, we set *α*_*i*_ = *α* for *i* = 1, 2, …, *h* and consider 2 cases: *α* = 1 or *α* = 5.

For the nonsymmetric Dirichlet distribution, we use:


$$\alpha_{i}=\left\{ \begin{array}{cc} 0.7\times w, & i=1\\ 0.3\times \frac{w}{h-1}, & i>1 \end{array}\right.$$

and again consider 2 cases: *w* = 5, 20. These combinations produce a diverse set of distributions (see Supplementary Material Section 1.1 and 1.2). The above combinatorial design produced 1,100 simulation combinations in total (see below). For each, we simulated from 10,000 to 100,000 genealogical classes using a discrete uniform probability distribution on the 6 distinct genealogical classes. As noted above, given a genealogical class, the diplotype of an individual is generated by simulating a pair of chromosomes (whether one or both chromosomes are pure or recombinant depends on the genealogical class). The simulator is available as an option in our program.

With 6 parameters (factors) and the number of corresponding levels for each (5 for *K*, 3 for *L*, 3 for *R*, 3 for *h*, 3 for *c*, and 4 for *α* and *ω*) the number of simulation combinations (when *L* > 1) is *C*_*L*>1_ = 5 × 2 × 3 × 3 × 3 × 4 = 1080. If *L* = 1, for each possible chromosome number (*K*) 4 ways exist to generate population haplotypes using Beta distributions (univariate cases of the Dirichlet distribution) with parameters *α*_1_ and *α*_2_:

(1)*α*_1_ = *α*_2_ = 1
(2)*α*_1_ = *α*_2_ = 5
(3)*α*_1_ = 0.7 × 5, *α*_2_ = 0.3 × 5
(4)*α*_1_ = 0.7 × 20, *α*_2_ = 0.3 × 20.

Thus, the number of combinations (for *L* = 1) is *C*_*L*=1_ = 5 × 4 = 20 and the total number of simulation combinations is *C*_*L*>1_ + *C*_*L*=1_ = 1, 080 + 20 = 1, 100.

We analyzed the statistical performance of Mongrail and NewHybrids (Anderson and Thompson 2002) for all the simulation combinations by computing for each simulated individual the posterior probabilities of belonging to each of the 6 genealogical classes. NewHybrids analyses multilocus genotypes and population allele frequencies, rather than haplotypes and haplotype frequencies. The genotypes are completely specified given the haplotypes. In our analyses, we ignore uncertainty of haplotype (or allele) frequencies, treating them as known. In this case, we do not require Markov Chain Monte Carlo (MCMC) sampling to compute the posterior probability of an individual belonging to each of the 6 different hybrid categories under the NewHybrids model, thus we implemented a version of NewHybrid without MCMC sampling to calculate posterior probabilities with known population allele frequencies. Our goal is to compare the 2 methods using the same assumptions. Avoiding MCMC allows a large-scale comparison without having to worry about whether NewHybrids MCMC analyses have converged. Uncertainty of haplotype or allele frequencies will be an additional source of variance for both estimators. It is straightforward to incorporate individual haplotype and population haplotype frequency inference into Mongrail and thus account for this source of uncertainty. For both methods, we considered a discrete uniform prior on the 6 distinct genealogical classes.

We performed a comparative analysis of statistical performance between Mongrail and NewHybrids using 5 different performance metrics:

(1)Accuracy of posterior probabilities
(2)Power to identify genealogical classes
(3)Posterior distribution of genealogical classes
(4)ROC curve analysis of power versus Type I error
(5)Sensitivity to biological and experimental parameters.

We describe the procedures used to evaluate the methods for each of these criteria in Supplementary Data Section 1.4.

### Coalescent simulation

The program *ms* (Hudson 2002) was used to simulate samples from 2 populations evolving according to a neutral Wright–Fisher model under various demographic histories. The program employs a structured coalescent model with recombination. Haplotype marker samples were simulated from the 2 populations (A and B) and used to estimate corresponding population haplotype frequencies. The diploid effective population size was *N*_0_ = 10, 000 for each population. We simulated 100 sampled chromosomes, each 1 Mb in length, for each population. The simulator is haploid so this corresponds to a sample of 50 diploid individuals from each population. We simulated 20 chromosomes for each diploid individual. The per-generation recombination probability over the entire chomosome was fixed to *r* = 0.01, thus the recombination rate was 1 cM/Mb. The population-scaled recombination rate parameter was then *ρ* = 4*N*_0_ × *r* = 40, 000 × 0.01 = 400. We assumed a symmetrical island model with *M* = 4*N*_0_*m*, where *m* is the fraction of each population made up of new migrants each generation. We chose 5 different values *M* = 0.1, 0.25, 1, 10, 100 to study the effects of migration on the performance of the 2 methods in identifying genealogical classes.

We used *ms* to generate gene trees representing the history of the sampled chromosomes. The *seq-gen* program (Rambaut and Grass 1997) was then used to simulate sequences on gene trees under a Jukes–Cantor mutation model. The population-scaled per-site mutation rate, *θ*, was assumed to be *θ* = 4*N*_0_*μ* = 0.00004, where *μ* = 10^−9^ is the mutation rate per generation. The program *snp-sites* (Page *et al.* 2016) was used to extract SNPs from simulated sequences into a variant call format (VCF) file. Subsequent processing and manipulations, such as extracting biallelic SNPs into separate VCF files for the 2 populations, were performed using BCFtools (Danecek *et al.* 2021). We chose a subset of *L* = 10 markers for each analysis such that the markers were approximately equidistant to each other and spanned the chromosome. Since we specified a recombination rate of 1 cM/Mb, the physical distance between the first and last marker was 1 Mb (the length of simulated chromosomes). Having obtained haplotype marker configurations for samples from both populations, unknown population haplotype frequencies were estimated using the Multinomial–Dirichlet posterior mean (see equation 1).

For each of 5 different values of *M*, we simulated 1,000 individuals for each of the 4 genealogical classes: purebred (model **d**), F1 (model **c**) hybrid, backcross (model **b**), and F2 (model **f**) hybrid. For brevity, we used only one backcross model (**b**) and one purebred model (**d**). Simulating a diploid individual is equivalent to generating a diplotype (a pair of haplotypes). Haplotypes required to form these individuals (belonging to any of the 4 genealogical classes) were simulated simultaneously with the population sample haplotypes that were generated under the structured coalescent process. The description of the procedure to generate diplotypes assigned to 1 of the 4 genealogical classes can be found in Supplementary Data Section 1.3.1.

For each simulated individual, we computed posterior probabilities under each of the 6 genealogical classes using either Mongrail or NewHybrids. We performed a comparative analysis of statistical performance between Mongrail and NewHybrids using 2 different performance metrics:

(1)Power to identify genealogical classes
(2)ROC curve analysis of power versus Type I error.

## Empirical analysis of spotted and barred owl hybridization

SO are native to the forests of western Northern America, mainly the Pacific Northwest, California and Mexico (Forsman *et al.* 1984; Anthony *et al.* 2006; Davis *et al.* 2016). BO are native to eastern North America but have expanded their range to the west coast of North America thus encroaching on the territory of the endangered spotted owl (Gutiérrez *et al.* 2007; Long and Wolfe 2019; Franklin *et al.* 2021), whose population is already in decline due to habitat loss caused by logging and wildfires (Clark *et al.* 2013; Ganey *et al.* 2017; Tempel *et al.* 2022). There are 3 recognized subspecies of the spotted owl ranging in distribution from British Columbia to Mexico: Northern spotted owl (NSO), California spotted owl (CSO), and Mexican spotted owl (MSO). The NSO and MSO have been listed as “threatened” under the Endangered Species Act since the early 1990s by the US Fish and Wildlife Service. As the 2 species can hybridize (sympatric populations of spotted and BO exist from British Columbia to southern California) (Hamer *et al.* 1994; Haig *et al.* 2004; Kelly and Forsman 2004), frequent hybridization may threaten the genetic integrity of the SO.

The study by Fujito *et al.* (2021) is the largest genomic study conducted on SO, BO, and their hybrids. They obtained sequences from spotted and BO sampled outside and across their hybrid zone in western Northern America. The sampling locations of all of the owls included in the study are presented in Supplementary Data Table S2. For County level information, see Fujito *et al.* (2021) (Supplementary Table S5). Fujito *et al.* (2021) improved upon a previously generated SO genome assembly (Hanna *et al.* 2017) (using data from 10× genomics and Bionano Genomics) and generated high-coverage (mean 31.70 × , ± 6.51) whole-genome sequence data from 51 owl samples consisting of 11 SO, 25 BO, 2 known hybrids (identified in Hanna *et al.* 2018) and 13 potential hybrids. The 51 owl samples included a female SO sample named Sequoia (Hanna *et al.* 2017) used for constructing the new and more contiguous reference genome assembly.

We used Mongrail to analyze the 51 owl samples, using the filtered variant call format (VCF) file available at https://trace.ncbi.nlm.nih.gov/Traces/sra/sra.cgi?analysis=SRZ190173. The dataset includes 17,385,299 biallelic SNPs across 82 large autosomal scaffolds and 8,543,351 of these had high-confidence genotype calls (*GQ* ≥ 40) in all individuals. We restricted our analyses to the 15 largest autosomal scaffolds and filtered out sites with any missing data. Here, we treat each scaffold as a chromosome. For each scaffold, we extracted the SO (10 individuals, excluding Sequoia) and BO (25 individuals) populations into 2 separate VCF files. Processing and manipulation of the high-throughput sequencing data was performed using BCFtools (Danecek *et al.* 2021). The unknown population haplotype frequencies (for SO and BO) were first estimated for each scaffold. Phased haplotype information is needed to estimate frequencies but the data were not phased for all SNPs. BEAGLE version 5.1 (Browning and Browning 2007) was used to phase each population separately for each scaffold. We chose 10 markers from each scaffold for the analysis. The distribution of markers across scaffolds varied (see Supplementary Section 2). The frequencies of phased haplotypes for the 2 populations were estimated using the Multinomial–Dirichlet posterior mean (see equation 1). In our model framework, BO is treated as Population A and SO as Population B. Thus the pure and hybrid classification of this owl dataset with its equivalent genealogical class based on our model:

- Model a - SO
- Model b - Backcross with BO (F1 × BO)
- Model c - F1 hybrid
- Model d - BO
- Model e - Backcross with SO (F1 × SO)
- Model f - F2 hybrid

The putative hybrids have an unusual plumage pattern making them difficult to distinguish from the western BO based purely on morphology. The main aim of this empirical analysis is to examine whether Mongrail can successfully place the samples into different genealogical classes, especially the hybrids. We also examine the effect of certain biological and experimental parameters on the power to identify genealogical classes, suggesting optimal ways to choose parameters (number of chromosomes or scaffolds and region size or expected recombination frequency/map length).

We perform 4 main analyses to examine the owl dataset which are as follows:

(1)Spatial variation of model posterior probabilities across scaffolds
(2)Effect of successive scaffold inclusion for varied map length
(3)Sensitivity of results to assumed recombination rate
(4)Assignment of 15 hybrids (13 putative and 2 known)

The procedures used to perform these 4 analyses are described in Supplementary Data Section 2.

## Results

### Comprehensive simulation

It is impossible to present an exhaustive summary of the properties of the methods when applied to all 1,100 combinations of simulation parameters considered in this paper. Instead, we give a general description of the most obvious patterns observed when applying each type of analysis to all the datasets and then provide specific examples for a subset of the combinations for which these patterns were most apparent. All the simulated datasets and scripts to perform the analyses are available at https://github.com/mongrail/simulations.

#### Accuracy of posterior probabilities

The general pattern observed across datasets when the analysis was done using Mongrail was that the average posterior probabilities matched the proportion of individuals correctly assigned to the specified genealogical class. However, the posterior probabilities and proportions typically did not match one another when using NewHybrids. The one exception was the case of a single locus per chromosome—in that case, the assumptions of NewHybrids (independent assortment of alleles across loci) were satisified and the posterior probabilities appeared correct. In other cases, posterior probabilities obtained using NewHybrids were higher than the proportion correctly assigned when posterior probabilities were high and were lower than the proportion correctly assigned when posterior probabilities were low.

**Fig. 3.**
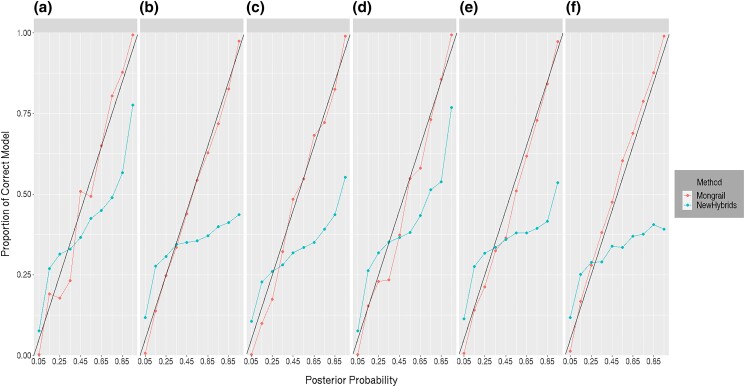
The proportion of correct assignments to genealogical classes (*y*-axis) for individuals binned according to the posterior probability of the genealogical class plotted against the midpoint posterior probability of each binning interval (*x*-axis). The 6 panels represent the 6 different genealogical classes. Results for posterior probabilities obtained using Mongrail are plotted in red and results for posterior probabilities obtained using NewHybrids are plotted in blue. If posterior probabilities match proportions the points should fall on a linear line with slope of unity (shown in black). Results are based on simulated data for 100,000 individuals using the simulation parameters: *K* = 20, *L* = 10, *R* = 50, *h* = 10, *c* = 0.1, *α* = 1. The 6 genealogical classes are as follows: **a**-pure population B, **b**-backcross with population A, **c**-F1 hybrid, **d**-pure population A, **e**-backcross with population B, **f**-F2 hybrid.

As an example, we generated 100,000 individuals under the set of simulation parameters: *K* = 20, *L* = 10, *R* = 50, *h* = 10, *c* = 0.1, *α* = 1. The Mongrail and NewHybrids programs were used to produce posterior probabilities for each distinct genealogical class for each individual. The posterior probabilities were binned into intervals as described in the Supplementary Data Section 1.4.1. The results are shown in Fig. 3. The proportion of individuals having the correct model is plotted against the midpoint posterior probability of the interval they were binned into. The results for Mongrail, shown in red, indicate a precise linear relationship with points lying very near the identity line; this is expected if the posterior probabilities are accurate. The results for NewHybrids, shown in blue, display an irregular curve with posterior probabilities higher than the proportion of correct genealogical classes when posterior probabilities are greater than about 40% and posterior probabilities lower than the proportion correct when posterior probabilities are lower than about 40%. Moreover, Mongrail performs well across all the genealogical classes (compare the 6 panels of Fig. 3 from left to right). The performance of the NewHybrids method appears nonuniform across the models, it seems to perform worse for models representing hybrids (**c, f**) and backcrosses (**b, e**) compared with the pure parental ones (**a, d**). Thus, even with a high recombination rate the NewHybrids composite likelihood method is too liberal. Since we are primarily interested in high posterior probabilities, it is a serious problem if estimates of high posterior probabilities are overconfident (the observed pattern fits our expectation that since NewHybrids is a composite likelihood method, it will tend to underestimate the uncertainty). See https://github.com/mongrail/simulations for additional examples of this behavior obtained using other combinations of parameters.

#### Power to identify genealogical classes

The general pattern observed across simulated datasets was that Mongrail typically placed higher posterior probability on the true genealogical class of an individual than did NewHybrids. NewHybrids always had more uniform probabilities among models but, although still worse, was closer in performance to Mongrail for individuals that were pure A or B (models **a** and **d**). In the case of 1 locus per chromosome, Mongrail and NewHybrids generate identical plots for posterior probabilities. This is expected because in this case NewHybrids is not a composite likelihood (all loci are unlinked).

**Fig. 4.**
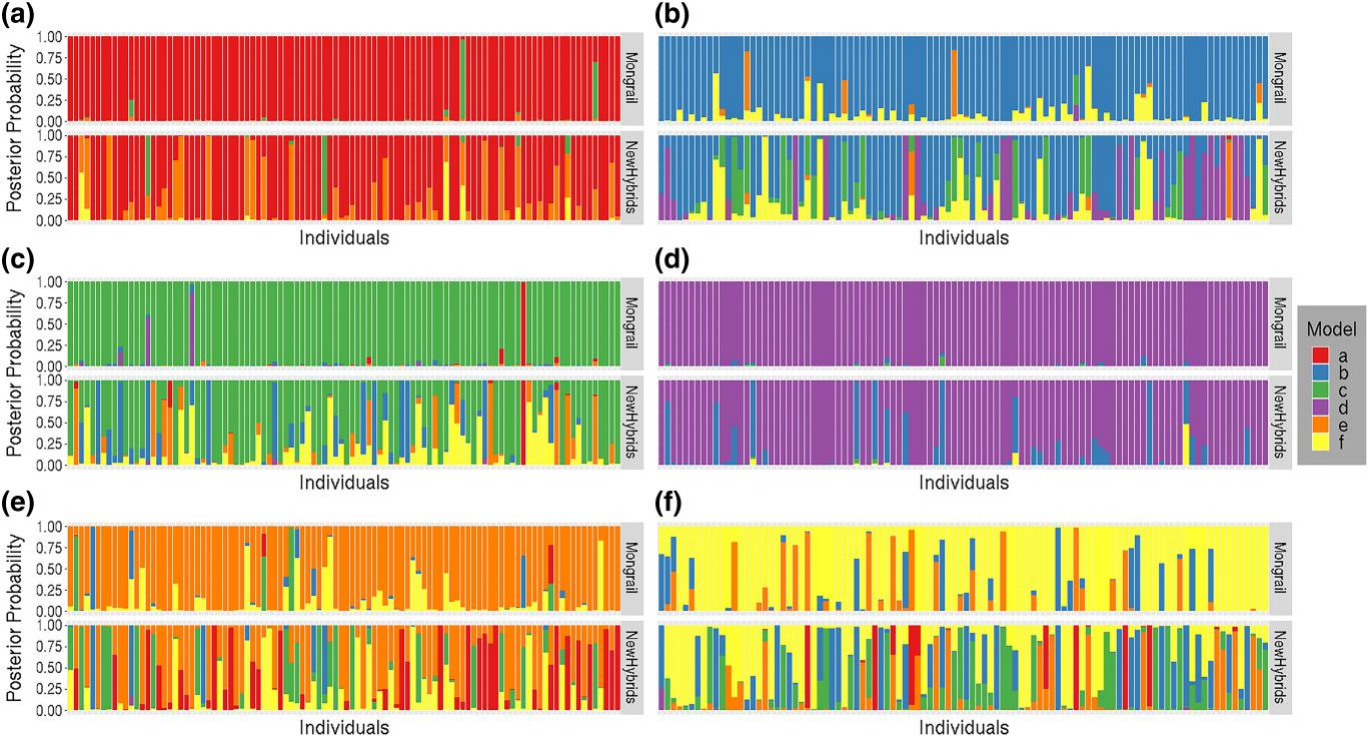
Distributions of posterior probabilities for random subsets of 100 individuals simulated under each of the 6 genealogical classes (plots labeled a–f). Posterior probabilities for Mongrail are shown in the top plot and for NewHybrids in the bottom plot. The posterior probabilities for different genealogical classes are represented by segments of different colors. The proportion of the stacked bar plot comprised of a particular color indicates the posterior probability of the model corresponding to that color. The following simulation parameters were used: *K* = 20, *L* = 10, *R* = 50, *h* = 5, *c* = 0.1, *α* = 1. The 6 genealogical classes are as follows: **a**-pure population B, **b**-backcross with population A, **c**-F1 hybrid, **d**-pure population A, **e**-backcross with population B, **f**-F2 hybrid.

Figure 4 shows the results for the particular simulation combination *K* = 20, *L* = 10, *R* = 50, *h* = 5, *c* = 0.1, *α* = 1. Due to space constraint we show the stacked bar plots for only 100 individuals from each of the 6 genealogical classes. The Mongrail method has high power in distinguishing the pure individuals (models **a, d**) and the F1 hybrids (model **c**)(top graph, panels labeled **a**, **d**, and **c** in Fig. 4). The posterior probability that Mongrail assigns to the correct genealogical class is for most individuals greater than 0.95. Even for the F2 hybrids (model **f**) and backcrosses (models **b, e**) support for the correct genealogical class increases substantially from the prior (uniform) to the posterior. When the posterior probability of belonging to the correct model **b** (backcross with pure population A) is less than 0.9, the remaining posterior probability is mostly assigned to the other hybrid and backcross categories. Similar patterns are observed for models **e** and **f** as we see a lot more variation (other colors which do not correspond to the true model).

Analyzing the same individuals using the NewHybrids method (bottom versus top graph) the posterior probabilities appear much more uniform across the genealogical classes with no particular genealogical class being well supported (this is particularly true for individuals that are hybrids or backcrosses; panels **b**, **c**, **e**, and **f**). These results suggest that the NewHybrids method may often produce poorly resolved genealogical classes for individuals when used with linked marker data. This is particularly the case when individuals are F2 hybrids or backcrosses.

#### Posterior distribution of genealogical classes

**Fig. 5.**
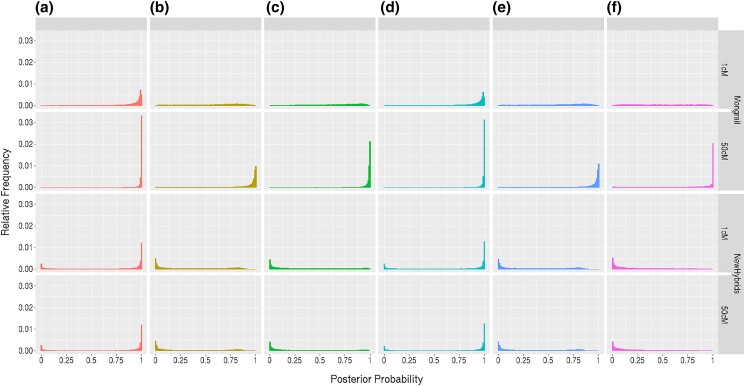
Histogram showing the relative frequency of the posterior probabilities obtained for the true model when analyzing simulated data using either the Mongrail (top 2 rows) or NewHybrids (bottom 2 rows) programs. The results are shown for data simulated using true models a–f (6 plots from left to right in each row). The plot is based on 100,000 individuals simulated using the following set of simulation parameters: *K* = 20, *L* = 10, *h* = 10, *c* = 0.1, and *α* = 1 with recombination frequencies on the interval of both *R* = 1 cM and *R* = 50 cM. The 6 genealogical classes are as follows: **a**-pure population B, **b**-backcross with population A, **c**-F1 hybrid, **d**-pure population A, **e**-backcross with population B, **f**-F2 hybrid.

This analysis examines the distribution of the posterior probability of a genealogical class when it is the true genealogical class for an individual (see description above). The general pattern across simulated datasets was that Mongrail tends to assign very high posterior probabilities only to the true model and very low posterior probabilities only to incorrect models. When the data are less informative the probabilities tend to be more uniform, resembling the prior. NewHybrids, on the other hand, less frequently assigns high probability to the true model and frequently assigns very low probabilities to the true model. These patterns are exemplified in Fig. 5. To create this figure, we generated 100,000 individuals under the following set of simulation parameters: *K* = 20, *L* = 10, *h* = 10, *c* = 0.1, and *α* = 1 with recombination frequencies on the interval of both *R* = 1 cM and *R* = 50 cM. The first row of the figure shows results for Mongrail with a relatively uninformative dataset (*R* = 1 cM). Even with low information, high posterior probabilities are frequently obtained for the true model when it is pure (model **a** or **d**) and for the other cases the probabilities are distributed quite uniformly and mostly intermediate. With *R* = 50 cM (second row of figure), high posterior probabilities occur much more commonly for the true model when it is any of the 6 models.

The results for the NewHybrids method are shown in rows 3 and 4 of Fig. 5. Since NewHybrids assumes unlinked markers, we expect the size of the region (frequency of recombination) will have no effect on the frequency distribution of posterior probabilities (the 2 rows with either *R* = 1 cM or *R* = 50 cM are identical). High posterior probabilities are obtained for the true model only when it is a pure population model (model **a** or **d**). For all the other models, the most frequent outcome is a very low posterior probability for the true model. Thus, NewHybrids has very low power to infer the true model when it is not a pure population model and will often exclude the true model, assigning very low probability to it.

#### ROC curve

**Fig. 6.**
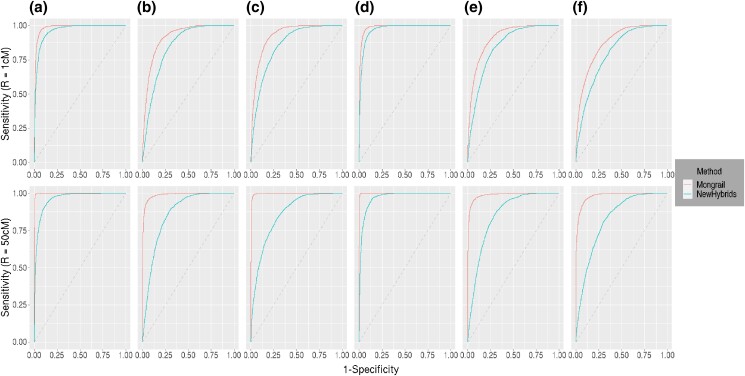
Reciever operator characteristics (ROC) curves for Mongrail (red line) and NewHybrids (blue line). The plot is based on 10,000 individuals simulated using parameters: *K* = 20, *L* = 5, *h* = 5, *c* = 0.1, and *α* = 1. The top row uses a smaller region (*R* = 1 cM) with a lower number of expected recombinations and the bottom row uses a larger region (*R* = 50 cM) with a higher number of expected recombinations. Results for the 6 genealogical classes (**a**–**f**) are shown from left to right in both rows. The 6 genealogical classes are as follows: **a**-pure population B, **b**-backcross with population A, **c**-F1 hybrid, **d**-pure population A, **e**-backcross with population B, **f**-F2 hybrid.

Here, we examine the relative tradeoff between power and type I error for each method using ROC curves (see description above). A method that has high power and low error should produce a curve that increases steeply and plateaus at a value approaching 1. The greater the area beneath the curve the better the performance. In general, Mongrail produces an ROC curve that strictly lies above the curve produced using NewHybrids when *L* > 1. As an example, we simulated 10, 000 individuals using the combinations of parameters: *K* = 20, *L* = 5, *h* = 5, *c* = 0.1, *α* = 1, and either *R* = 1 or *R* = 10. The results are shown in Fig. 6.

The Mongrail inference method outperforms NewHybrids across all the 6 genealogical classes. In the case of a recombination frequency of 1cM, the difference between the 2 methods is least pronounced with either pure population A (model **d**) or B (model **a**) ancestry. As the recombination frequency increases from 1 cM to 50 cM, the ROC curve for the Mongrail method improves, approaching the top left corner, whereas the curve for NewHybrids is virtually unchanged. The NewHybrids method assumes unlinked markers and thus we expect its ROC curve to be unaffected by recombination (compare the blue curves on the top row to the bottom one).

#### Sensitivity to biological and experimental parameters

**Fig. 7.**
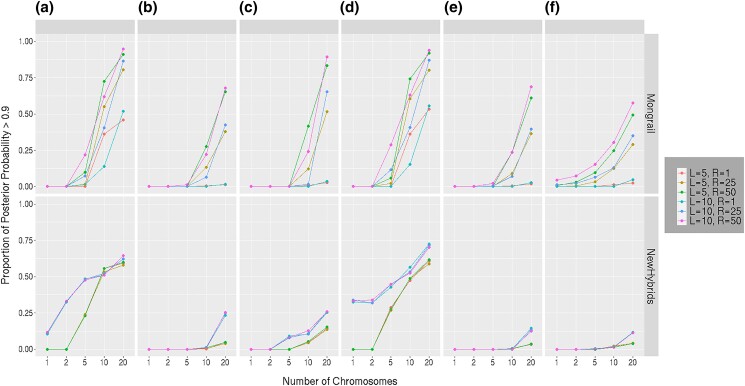
Proportion of simulated individuals with posterior probabilities for the correct genealogical class greater than 0.9 (*y*-axis) versus the number of chromosomes sampled per individual (*x*-axis). Different colored curves are plotted for 6 different combinations of numbers of loci and expected recombination rates (in cM). The numbers of loci and expected recombination frequency is denoted by *L* and *R*, respectively. The results for Mongrail are shown in the top row and for NewHybrids in the bottom row. Results for the 6 genealogical classes that individuals were simulated under are given from left to right in each row. The 6 genealogical classes are as follows: **a**-pure population B, **b**-backcross with population A, **c**-F1 hybrid, **d**-pure population A, **e**-backcross with population B, **f**-F2 hybrid.

Here, we examine the relative influence of 3 parameters: number of chromosomes, number of loci, and recombination frequency on the power of the methods to identify genealogical classes (using a posterior probability of 0.9 as a threshold for classifying individuals). Other parameters were held constant (see above). Figure 7 shows the proportion of cases with posterior probability for the true genealogical class greater than 0.9 (*y*-axis) as a function of the number of chromsomes (*x*-axis) for different combinations of number of loci and expected recombination rate for the Mongrail method (top row) and for NewHybrids (bottom row). Some interesting patterns emerge from the multi-line plot for Mongrail (Fig. 7). Each colored line in Fig. 7 shows that as the number of chromosomes considered increases, the proportion of cases where the posterior probability is above 0.9 increases as well. The increase is particularly large when the number of chromosomes increases from 5 to 20. This is true across all genealogical classes. However, as observed for other metrics the genealogical classes **a, c,** or **d** receive higher posterior probabilities for the correct class when compared with the other 3 genealogical classes, likely because the data are more informative in these cases. Another striking trend is apparent across all the genealogical classes, the proportion above 0.9 changes little with an increase in number of markers but steadily increases with increasing recombination frequency from 1cM to 50 cM. In summary, increasing the number of chromosomes or recombination frequency has a large effect on the posterior probabilities (the former having greatest effect) whereas increasing the number of loci has little effect. With only 1 or 2 generations of mating one expects few recombination events, even on large intervals and so a small number of markers are sufficient to capture the available information from the data. Because chromosomes undergo independent assortment adding additional chromosomes has a much greater effect on power. Similarly, increasing the size of a region of chromosome (even with a fixed total number of markers) increases the chances of observing recombination events and also increases power.

We analyze the same simulated dataset using the NewHybrids method (bottom row of Fig. 7) for a side-by-side comparison of the effects of the 3 factors on the 2 competing methods. As expected, we see that the proportions for *R* = 1, 25, and 50 merge, regardless of the number of loci. Because NewHybrids is a composite likelihood method, we expect that recombination frequency should have little or no effect on the posterior probabilities and this is indeed the case. We find that increasing number of chromosomes increases the proportion of cases for which the posterior probability is greater than 0.9 but the changes are pronounced only for the pure (**a, d**) and F1 hybrid (**c**) individuals. In fact, an increase in the number of loci (from 5 to 10) increases the proportion only for these 3 classes. Only when the number of chromosomes is *K* = 20, is there is a difference in the proportions (at *L* = 5, 10) for the remaining genealogical classes (**b,e,f**).

### Coalescent simulation

We performed simulation analyses for *M* = 0.1, 0.25, 1, 10, 100 and 4 genealogical classes: purebred (model **d**), F1 (model **c**) hybrid, backcross (model **b**), and F2 (model **f**) hybrid. For brevity, we describe the general pattern observed across the analyses and present details only for a subset of specific illustrative cases. Results for the complete set of simulated datasets and scripts to perform the analyses are available at https://github.com/mongrail/simulations.

#### Power to identify genealogical classes

The general pattern observed across simulated datasets for genealogical classes purebred (model **d**) and F1 hybrids (model **c**) with *M* ≤ 1 was that both Mongrail and NewHybrids performed well in identifying true genealogical classes. Both methods produced high posterior probabilities for the true genealogical class with neither obviously superior. However, when considering all 4 genealogical classes (models **d**,**c**,**b**,**f**) with *M* > 1, performance of Mongrail in identifying the true genealogical class is typically better than NewHybrids, although the power to distinguish correct genealogical classes diminishes for both methods as *M* increases.

**Fig. 8.**
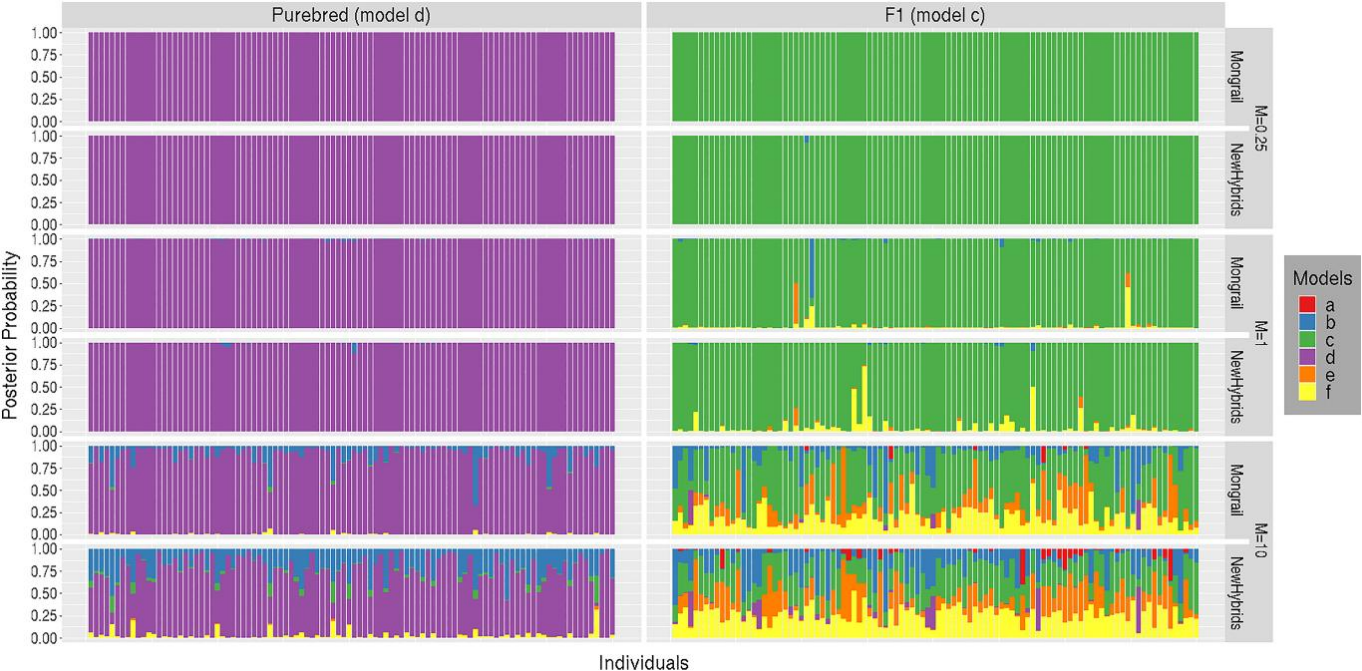
Distributions of posterior probabilities for random subsets of 100 individuals simulated (through a structured coalescent process) under each of the 2 genealogical classes (plots labeled d and c) for 3 values of migration parameter *M* = 0.25(1st, 2nd rows), 1(3rd, 4th rows), 10(5th, 6th rows). For each value of *M*, posterior probabilities for Mongrail are shown in the top plot and for NewHybrids in the bottom plot. The posterior probabilities for different genealogical classes are represented by segments of different colors. The proportion of the stacked bar plot comprised of a particular color indicates the posterior probability of the model corresponding to that color. The 6 genealogical classes are as follows: **a**-pure population B, **b**-backcross with population A, **c**-F1 hybrid, **d**-pure population A, **e**-backcross with population B, **f**-F2 hybrid.

**Fig. 9.**
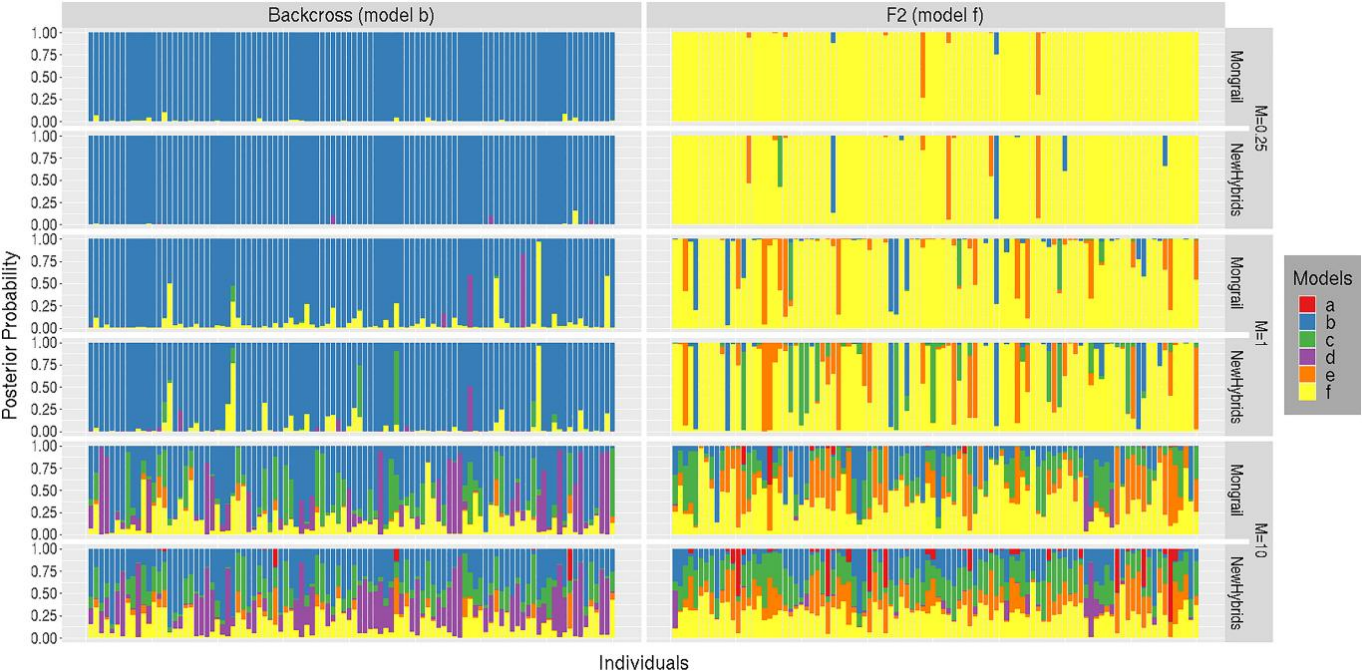
Distributions of posterior probabilities for random subsets of 100 individuals simulated (through a structured coalescent process) under each of the 2 genealogical classes (plots labeled b and f) for 3 values of migration parameter *M* = 0.25(1st, 2nd rows), 1(3rd, 4th rows), 10(5th, 6th rows). For each value of *M*, posterior probabilities for Mongrail are shown in the top plot and for NewHybrids in the bottom plot. The posterior probabilities for different genealogical classes are represented by segments of different colors. The proportion of the stacked bar plot comprised of a particular color indicates the posterior probability of the model corresponding to that color. The 6 genealogical classes are as follows: **a**-pure population B, **b**-backcross with population A, **c**-F1 hybrid, **d**-pure population A, **e**-backcross with population B, **f**-F2 hybrid.

Figure 8 shows results for purebreds (model **d**, first column) and F1 hybrids (model **c**, second column). Figure 9 shows results for backcrosses (model **b**, first column) and F2 hybrids (model **f**, second column). Both figures show results for a range from relatively low to high values of migration (*M* = 0.25, 1, 10). The stacked bar plots for only 100 representative individuals from each of these 4 genealogical classes are shown due to space constraints. For all 4 genealogical classes (Figs. 8 and 9) when *M* ≤ 1 (first 4 rows), Mongrail (first and third row), and Newhybrids (2nd and 4th row) perform similarly well, both placing high posterior probability on the true genealogical classes. For most individuals, the posterior probability assigned to the true genealogical class by both methods is greater than 0.9. More probability associated with incorrect models (colors not corresponding to the true model) is evident for backcrosses and F2 hybrids in Fig. 9 compared with the pure and F1 genealogical classes (Fig. 8). For *M* > 1 (last 2 rows), both methods show more uncertainty across all 4 genealogical classes but Mongrail appears to perform better on average. The other general pattern observed is that both methods struggle to resolve the correct genealogical class as model complexity increases (from purebred to F1 hybrid in Fig. 8 or from backcross to F2 hybrid in Fig. 9) especially when the migration rate is high (*M* = 10). Difficulty increases with an increase in model complexity (models **d**,**c**,**b**,**f**) across all values of *M*. This is evident from the more uniform distribution of posterior probabilities across genealogical classes. For extremely high migration between the 2 populations, NewHybrids performs less well in resolving genealogical classes by comparison with Mongrail. This is especially true for F1 or F2 hybrids or backcrosses.

#### ROC curve

**Fig. 10.**
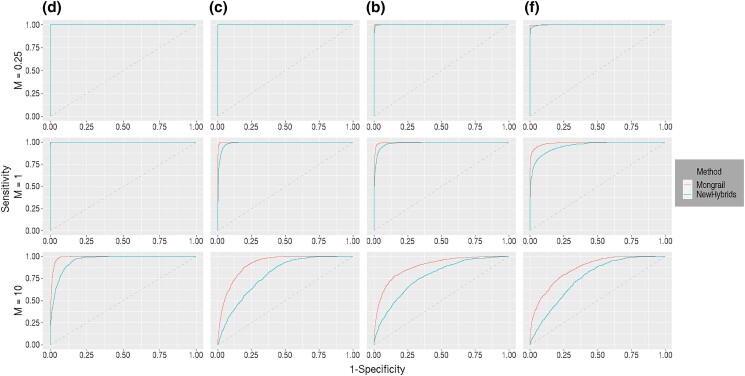
Reciever operator characteristics (ROC) curves for Mongrail (red line) and NewHybrids (blue line). The plot is based on 1000 individuals simulated under each of the 4 genealogical classes (**d,c,b,f**) through a structured coalescent process. The first, second, and third row corresponds to migration parameter with values *M* = 0.25, 1, and 10, respectively. Results for the 6 genealogical classes (**d,c,b,f**) are shown from left to right in all 3 rows. The 6 genealogical classes are as follows: **a**-pure population B, **b**-backcross with population A, **c**-F1 hybrid, **d**-pure population A, **e**-backcross with population B, **f**-F2 hybrid.

The relative tradeoff between power and type I error was analyzed for each method using ROC curves for the datasets simulated under a structured coalescent process. The greater the area beneath the curve, the better the performance. In general, for all *M* ≥ 1, Mongrail produces an ROC curve that lies strictly above the curve produced by NewHybrids across all 4 genealogical classes (models **d**,**c**,**b**,**f**). We present the results for 1000 individuals simulated under each of the 4 genealogical classes for *M* = 0.25, 1, 10 in Fig. 10.

Mongrail outperforms NewHybrids across all 4 genealogical classes when *M* ≥ 1. The performance difference between the 2 methods increases with model complexity, with *M*( ≥ 1) fixed. As *M* increases, the difference between the 2 methods gets more pronounced for all the 4 genealogical classes. When *M* < 1 for the purebred (model **d**) and F1 hybrid (model **c**), the 2 curves overlap with no visible difference between methods. When *M* < 1 for the backcross (model **b**) and F2 hybrid (model **f**), although the Mongrail curve lies above the NewHybrids curve, the difference is negligible.

### Empirical analysis of spotted and barred owl hybridization

All analyses were performed using the 50 owl samples described in Table S2 (Supplementary material) excluding Sequoia. For brevity, detailed results are presented for only 5 individuals. The 5 owls were chosen to be representative of the 5 observed categories genetically identified by Fujito *et al.* (2021). The categories along with the sample names are presented in Table 1. Phased haplotypes were not available for these owls. Putative “pure” individuals ZRH625 (SO) and ZRHG101 (BO) were phased using BEAGLE version 5.1 (Browning and Browning 2007). For the remaining putative hybrid, or backcross, individuals inferences were averaged over the probability distribution of possible phase resolutions using equations 2 and 3.

**Table 1.** Details for 5 individuals (out of the 50 owls) chosen for detailed analysis. Primary and genetic identifications are from Fujito *et al.* (2021).

Primary identification	Genetic identification	Sample names
spotted owl (SO)	SO	ZRH625
barred owl (BO)	BO	ZRHG101
putative hybrid	BO	CYWC009
known hybrid	backcross (F1 × BO)	ZRH607
known hybrid	F1 hybrid (F1)	ZRH962

#### Spatial variation of model posterior probabilities

**Fig. 11.**
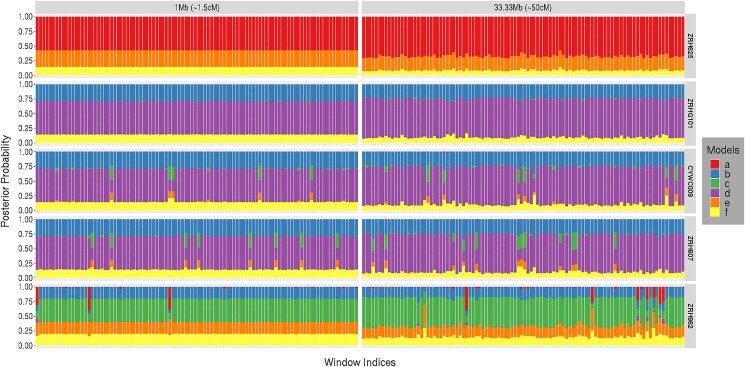
Distribution of posterior probabilities is constructed for 5 owl samples (ZRH625, ZRHG101, CYWC009, ZRH607, and ZRH962) for 100 windows (set of *L* = 10 markers), where the initiating markers are evenly spaced across the largest scaffold Super-Scaffold_7 (size 72.11 Mb). The right column uses a larger region (33.33 Mb equivalent to *R* = 50 cM) of the scaffold and the left column uses the same initiating markers but of smaller length (1 Mb equivalent to *R* = 1 cM). Six different colors are used to represent the 6 different genealogical classes. The proportion of the stacked bar plot comprised of a particular color indicates the posterior probability of the model corresponding to that color. The primary and genetic identification information about the 5 owl samples are provided alongside the sample identifiers. A recombination rate of *r* = 1.5 cM/Mb is assumed for this plot. The 6 genealogical classes are as follows: **a**-pure population B, **b**-backcross with population A, **c**-F1 hybrid, **d**-pure population A, **e**-backcross with population B, **f**-F2 hybrid. In our model framework, BO is treated as population A and SO as population B.

In this analysis, we examine how posterior probabilities vary across a scaffold. We also examine the effect of the size of a window (map length) on the distribution of posterior probabilities. Figure 11 shows the distribution of posterior probabilities across the largest scaffold (Super-Scaffold_7) of length 72.11 Mb. The distribution of the posterior probability on the 6 genealogical classes (denoted by each bar) is highly consistent across the entire scaffold for all the samples. Increasing map length from *R* = 1.5 cM (left) to *R* = 50 cM (right) increases the posterior probabilities for the proposed genealogical class for the pure individuals and the F1 hybrid. The pattern in the putative hybrid (row 3) and backcross (row 4) is less clear. For these 2 owl samples, there is a slight increase in the posterior probability for the pure barred owl genealogical class (model **d**) but also increased support for F1 hybrid (model **c**) in some regions. Such patterns might suggest more ancient hybridization not included in our model. There is also more variation in the posterior probabilities for the larger window size along the scaffold, likely because the markers are spread over a larger region thus increasing the chances of recombination. The stacked bar plots for the putative hybrid and the backcross individuals look very similar suggesting that distinguishing the hybrids may be quite difficult when using a small region of a single chromosome. Distinguishing the genealogical classes of these 2 hybrids may not be possible based on a single scaffold.

#### Effect of successive scaffold inclusion for varied map length

This analysis evaluates the cumulative effect of the number of scaffolds and of map length on the posterior probability for each genealogical class. Another aim is to examine whether the genealogical class with highest posterior probability for an individual analyzed using Mongrail matches the genetically identified category of Fujito *et al.* (2021) for that individual.

**Fig. 12.**
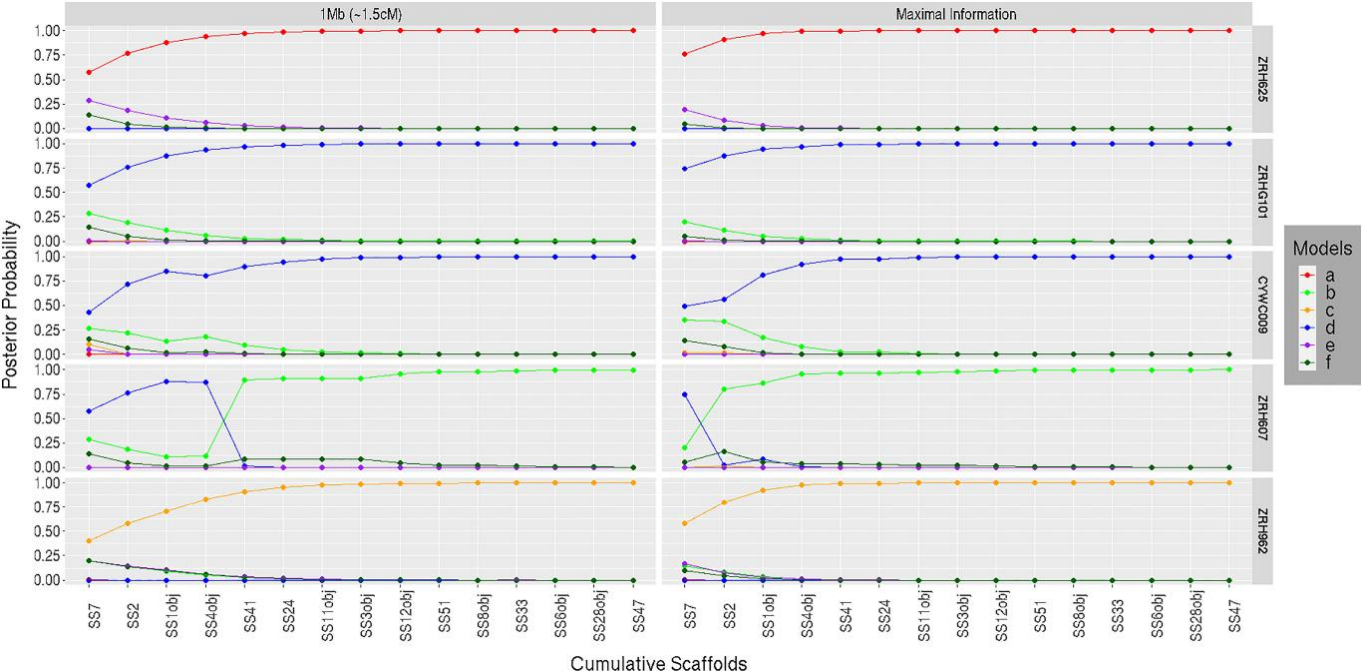
Posterior probability plotted against the cumulative number of scaffolds for 5 owl samples (ZRH625, ZRHG101, CYWC009, ZRH607, and ZRH962). Different colored lines are plotted for 6 different genealogical classes. Moving along the *x*-axis, the scaffolds (SS stands for Super-Scaffold) are arranged from the largest to smallest and the number of scaffolds (*K*) increases from 1 to 15. The right column referred to as the “maximally informative” case considers very large map length (its equivalent physical length is almost equal to the length of the scaffold) for every scaffold and a set of *L* = 10 markers are chosen from the middle of each scaffold where the markers are almost equidistant from each other. The left column considers a smaller region (1 Mb equivalent to *R* = 1.5 cM) for each scaffold with *L* = 10 markers with the same initiating markers chosen previously (for the “maximally informative” case). The primary and genetic identification information about the 5 owl samples are provided alongside the sample identifiers. A recombination rate of *r* = 1.5 cM/Mb is assumed for this plot. The 6 genealogical classes are as follows: **a**-pure population B, **b**-backcross with population A, **c**-F1 hybrid, **d**-pure population A, **e**-backcross with population B, **f**-F2 hybrid. In our model framework, BO is treated as population A and SO as population B.

To examine the cumulative effect of adding scaffolds, we used the 15 largest scaffolds, which were sequentially added in descending order by size. In Fig. 12, as we move from left (Super-Scaffold_7) to right (Super-Scaffold_47) on the *x*-axis, the number of scaffolds denoted by *K* increases from 1 to 15. The general pattern observed is that the posterior probability for genealogical classes **a, d**, and **c** increases monotonically (asymptotically approaching 1) as the number of scaffolds is increased for the pure spotted owl (ZRH625), pure barred owl (ZRHG101) and F1 hybrid (ZRH962). Across the 3 samples, in the “maximally informative” case, the posterior probability of the “preferred” model exceeds 0.9 when *K* = 3 and exceeds 0.99 when *K* = 5. The “maximally informative” case (right) occurs when we choose map lengths (for each scaffold) such that the equivalent physical length is near the length of the scaffold. With a smaller region (lower expected recombination frequency) of *R* = 1.5 cM (left), the posterior probability of the genealogical class **a** (or, **d**) exceeds 0.9 when *K* = 4 and exceeds 0.99 when *K* = 7 for spotted owl (or, barred owl), respectively. For the F1 hybrid at *R* = 1.5 cM, the posterior probability of genealogical class **c** exceeds 0.9 when *K* = 5 and exceeds 0.99 when *K* = 9. These results suggests that either increasing the number of scaffolds or increasing the within-scaffold region size increases information, but additional scaffolds have a greater effect. The smaller the region (lower the expected recombination frequency) the higher the number of scaffolds (*K*) needed for the posterior probability of the “preferred” model to approach 1.

For the putative hybrid, even in the “maximally informative” case, more scaffolds are needed by comparison with the pure barred owl for the genealogical class **d** to become the “preferred” one. The posterior probability of genealogical class **d** exceeds 0.9 when *K* = 5 and exceeds 0.99 when *K* = 8. A similar posterior probability pattern is observed for *R* = 1.5 cM with an increasing number of scaffolds. Though the “preferred” model is the same (**d**) for both the samples (pure barred owl and putative hybrid) a striking contrast is observed between posterior probability plots for the 2 individuals. This difference may suggest why the putative hybrid individual was difficult to differentiate from a pure barred owl based solely on morphology (it had an unusual plumage pattern). It is possible that this individual is descended through a backcross greater than 2 generations ago. Distinctive patterns of posterior probability are observed in the putative backcross individual for both choices of region size (expected recombination frequency). Two genealogical classes **b** and **d** seem to be competing for relative support, with **d** preferred initially. As the number of scaffolds is increased, a point is reached where the trend of the 2 line plots is reversed and genealogical class **b** is increasingly favored, ultimately emerging as the “preferred” model. The main difference between the scaffold addition plot for the 2 different region sizes (*R* = 1.5 cM and “maximally informative”) is that the transition to the asymptotically preferred model takes place with fewer scaffolds in the “maximally informative” case (the transition occurs as *K* = 1 increases to *K* = 2 in the “maximally informative” case versus as *K* = 4 increases to *K* = 5 in the *R* = 1.5 cM case). The posterior probability for genealogical class **b** exceeds 0.99 when *K* = 10 in the “maximally informative” case but more scaffolds (*K* = 13) are needed for the posterior probability to exceed 0.99 when the map length is *R* = 1.5 cM.

In conclusion, the “preferred” genealogical class for each owl sample matches the previously genetically identified class indicating that Mongrail is successful in inferring genealogical classes irrespective of the choice of region size (*R* = 1.5 cM or “maximally informative”). The larger “maximally informative” region size ultimately supported the same genealogical classes as the smaller region but converged to a high posterior probability with fewer scaffolds. This suggests that a researcher has some flexibility to design genomics experiments with different numbers of scaffolds and region sizes according to the limitations imposed by their budget or study organism.

#### Sensitivity of results to assumed recombination rate

**Fig. 13.**
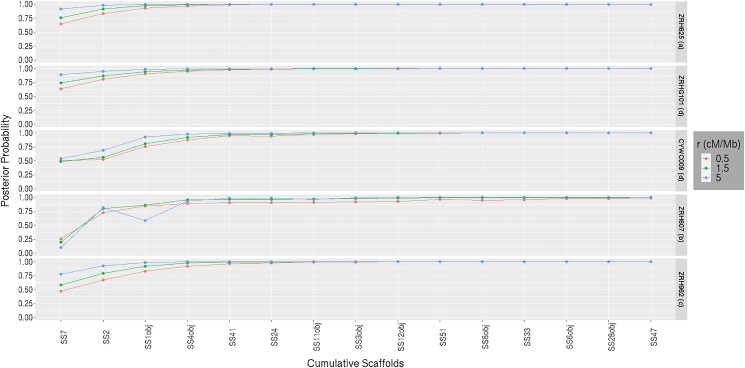
Posterior probability of the “preferred” model is plotted against the cumulative number of scaffolds for 5 owl samples (ZRH625, ZRHG101, CYWC009, ZRH607, and ZRH962) at different levels of recombination rate (*r* = 0.5, 1.5, 5 cM/Mb). Different colored plots denote different values of recombination rate. A genealogical class (or, model) is said to be “preferred” if its posterior probability approaches 1 while the posterior probability for the rest concentrates around 0 with large number of scaffolds. The “preferred” model is shown in parenthesis placed beside the sample names. Moving along the *x*-axis, the scaffolds (SS stands for super-scaffold) are arranged from the largest to smallest and as we move from left to right the number of scaffolds (*K*) increases from 1 to 15. A set of *L* = 10 markers are chosen from the middle of each scaffold where the map length for every scaffold is selected in such a way that its equivalent physical length is almost equal to the length of the scaffold. The markers are almost equidistant from each other. The primary and genetic identification information about the 5 owl samples are provided alongside the sample identifiers.

The Mongrail method requires that recombination rate be known (in units of cM/Mb). For the Spotted and BO species, direct estimates of recombination rates across the genome (from pedigree analysis for example) are unavailable. Instead, we used a recombination rate of 1.5 cM/Mb based on the average recombination rate of the zebra finch. Here, we examine the sensitivity of the results generated by Mongrail to assumptions about the recombination rate. Specifically, we examined the influence of different values of recombination rate on the inferred genealogical class. Figure 13 shows the posterior probability for the “preferred” model as a function of the number of scaffolds (*K*) at different values of recombination rate for each owl sample. A model is “preferred” if its posterior probability approaches one as the value of *K* increases. The general pattern observed across all the owl samples is that the “preferred” models for the spotted owl, barred owl, putative hybrid, backcross and F1 hybrid when all scaffolds are used are, respectively, **a** (Row 1), **d** (Row 2), **d** (Row 3), **b** (Row 4), and **c** (Row 5) regardless of the recombination rate that was used. Thus, for the owl dataset, varying the recombination rate over a broad range does not change the genealogical classes inferred by Mongrail. The same genealogical class is likely to be identified even if the recombination rate is badly mis-specified and our extrapolation of the recombination rate from another species is unlikely to be misleading for this dataset.

#### Assignment of 15 hybrids (13 putative and 2 known)

Here, we examine whether the Mongrail method produced posterior probabilities for “preferred” models high enough to allow classification of a hybrid sample. Table 2 shows that if 0.99 is chosen as the threshold value to make a decision, we were able to make an assignment call for all hybrids except one (TLW519). The posterior support for the one unassigned hybrid sample TLW519 is still very high (0.96) although below the threshold (0.99) and it too would be assigned if a lower threshold (such as 0.95) were used. Based on our model framework, genealogical class **d**, **c**, and **b** refers to a pure barred owl, a F1 hybrid and a backcross with barred owl, respectively. We see that the inferred genealogical class for each sample matches the prior genetic classification suggesting that Mongrail provides reasonable results that align with previous conclusions (analysis with NewHybrids produces similar results, see Supplementary Table S3).

**Table 2.** Table showing the assignment call for all 15 hybrid owl samples. The primary and genetic identification were provided by Fujito *et al.* (2021). Assuming a recombination rate of 1.5cM/Mb and considering the “maximally informative” case (the map length for each scaffold is chosen in such a way that its equivalent physical length is almost equal to the length of the scaffold) for all 15 scaffolds, the “preferred” model along with its posterior probability is presented in the table. Based on our model framework, genealogical class **d**, **c**, and **b** refers to a pure barred owl, a F1 hybrid and a backcross (with barred owl), respectively.

Sample names	Primary identification	Genetic identification	Posterior probability	Preferred model
TLW521	Unknown	Barred owl	1	d
TLW532	Unknown	Barred owl	1	d
AFRD90	Unknown	Barred owl	1	d
CYWC009	Unknown	Barred owl	1	d
1957-00137	Unknown	F1	1	c
1957-00240	Unknown	F1	1	c
1957-00243	Unknown	F1	1	c
LCW1363	Unknown	F1	1	c
LCW1383	Unknown	F1	1	c
ZRH600	Unknown	F1	1	c
ZRH610	Unknown	F1	1	c
ZRH962	Known hybrid	F1	1	c
TLW519	Unknown	Backcross	0.961	b
TLW528	Unknown	Backcross	1	b
ZRH607	Known hybrid	Backcross	0.999	b

## Discussion

In this article, we developed a full-likelihood Bayesian method to identify species hybrids, explicitly modeling recombination, using population genome sequence data (multiple biallelic SNP markers). We compute the posterior probability of an individual belonging to different genealogical classes (pure population, F1 or F2 hybrid, or backcross). Hybridization is an important evolutionary process influencing biodiversity and impacting diversity generating processes such as speciation (Abbott *et al.* 2013). Mongrail should thus be particularly useful to researchers in the fields of conservation biology or population management. In particular, identifying hybrids is a first step in studying various consequences of natural hybridization such as hybrid inviability, introgression between species, and so on (Arnold 1992). The method developed here offers an improvement over the hybrid inference method of Anderson and Thompson (2002) for the analysis of population genomic data by explicitly modeling linked markers. Rapid progress in sequencing technologies has produced datasets with hundreds of thousands of linked loci, clearly violating this assumption. Treating linked loci as independent reduces NewHybrids to a composite likelihood method, potentially leading to overconfidence and/or inaccuracy in identifying hybrids.

We considered a simple model of recombination which closely mimics the population genetic process. This allowed us to build a full-likelihood method with linked markers without the need to use MCMC, reducing the burden of heavy computational cost and the risk of nonconvergence. We compared the performance of Mongrail and NewHybrids by performing 2 extensive simulation studies. The first type, a comprehensive simulation study, aimed at investigating the 2 inference methods under a broad range of haplotype frequency distributions. The second type, based on a structured coalescent model was performed to generate biologically realistic linkage disequilibrium patterns among haplotypes from the source populations. The simulator generates a pair of haplotypes (a diplotype) for an individual from 2 populations assuming linkage and recombination in the formation of hybrids. Simulated data were analyzed to compute the posterior probabilities of genealogical classes using both Mongrail and NewHybrids. This allowed us to evaluate their relative statistical performance in distinguishing the different genealogical classes.

The comprehensive simulation study demonstrated the improved performance of Mongrail over NewHybrids using varying levels of biological and experimental parameters such as number of chromosomes, number of loci, expected recombination frequency (in cM), etc. The simulation studies indicated that Mongrail was often able to correctly identify the genealogical classes with high certainty, whereas NewHybrids more often failed to infer the correct genealogical class, especially in the case of hybrids or backcrosses. Even with high values of expected recombination frequency, NewHybrids often overestimated the posterior probabilities (as expected in a composite likelihood). With only 2 generations of mating, few recombinations are expected over the entire chromosome, violating the assumption of the Anderson and Thompson (2002) method that markers are unlinked.

The comprehensive simulation study also suggested that for, both methods, increasing the number of chromosomes has a large effect on the power to infer the correct genealogical class, whereas increasing the number of markers has little effect. Increasing the map-length of the chromosome also greatly increases power by increasing the expected number of recombinations. One outcome of this is that relatively few markers provide sufficient power, reducing the potential computational cost incurred due to a large number of linked loci. Currently, our program implementing this algorithm (Mongrail) allows only 10 markers per chromosome, although the statistical model allows for an arbitrary number of markers. This limitation of the current program does not negatively impact our empirical analysis since we can utilize the power of increased numbers of chromosomes. Many diploid species have sufficient numbers of chromosomes to render the method powerful. The computational complexity increases only linearly in the number of chromosomes.

The coalescent simulation study evaluated the performance of the 2 methods in inferring individual genealogical classes with varying levels of migration between populations. We considered a wide range of migration, *M* ∈ {0.1, 0.25, 1, 10, 100}. Applying Wright’s approximate formula for expected *F*_*ST*_ (fixation index), *F*_*ST*_ = 1/(4*N*_0_*m* + 1) = 1/(*M* + 1), these values correspond to *F*_*ST*_ ∈ {0.91, 0.8, 0.5, 0.091, 0.0091}. Low values of migration translate to a high value of *F*_*ST*_, indicating greater genetic differentiation between the 2 populations and vice versa. This simulation study suggested both methods perform well inferring purebreds for low values of migration (*M* ≤ 1 equivalent to *F*_*ST*_ ≥ 0.5). But with an increase in migration, Mongrail performs better in distinguishing the genealogical classes on average compared with Newhybrids. As model complexity increases (from F1 hybrid to backcross to F2 hybrid) the performance of both methods declines. In general, Mongrail is more effective in distinguishing hybrids and backcrosses compared with Newhybrids under the simulation conditions we examined, even when genetic differentiation between populations is low.

We applied Mongrail to a previously published whole-genome sequence dataset consisting of SO, BO, and their hybrids. Mongrail was able to infer genealogical classes for all the putative hybrids as well as the purebreds with high posterior probability. This was true despite using only 10 markers from each of 15 largest autosomal scaffolds. The markers were spread evenly across the entire length of scaffold to attain maximum information from the data. No prior information were available on the hybridization rates or differential fitnesses among the 6 genealogical classes, thus a discrete uniform prior on the classes was used. We also assumed a uniform recombination rate on each scaffold—as no prior information was available for the owl dataset—and extrapolated the specific value from another species, the zebra finch. Mongrail is able to accomodate variable recombination rates if such information is available. The genealogical classifications did not change when other much higher (or lower) rates were used, suggesting that for many species a rough estimate of the recombination rate should be sufficient for the use of Mongrail.

The model underlying Mongrail assumes random mating (Hardy–Weinberg equilibrium), and the method requires phased diplotypes and specified haplotype frequencies for the 2 populations. Many genomic datasets are comprised of unphased genotype data and the population haplotype frequencies are usually unknown. In our study, we phased the pure individuals using BEAGLE version 5.1 (Browning and Browning 2007) on each population separately. With advances in sequencing technologies complete phased data may be common in future, thus eliminating the need for phase inference. Population haplotype frequencies were estimated using the Multinomial–Dirichlet posterior mean of the reference samples. An alternative would be to allow for uncertainties by integrating over the posterior density. An advantage of our approach is that we calculate the likelihood of hybrid individuals without assuming phase, by integrating over all compatible haplotypes thus taking into account the uncertainties.

A current limitation of Mongrail, is that it only allows biallelic SNP loci. The vast majority of SNP loci in most species are biallelic, so plenty of biallelic SNPs are available for use rendering this constraint largely unimportant. Nonetheless, the method can be easily extended to multiple alleles by redefining the bit operations of the program. An assumption of the current method is that hybridization between the 2 species (populations) occurred within the last *n* = 2 generations. Therefore, the extent to which many generations of backcrossing (*n* > 2) affects our inference method is unknown. Since we suspect that individuals resulting from many generations of unidirectional backcrossing may resemble purebreds genetically this potentially limits the scope of our method. However, information probably dissipates quickly with additional generations of hybridization and the number of possible models quickly increases—there could also be identifiability issues. We suggest that it is sensible to focus exclusively on identifying recent hybrid ancestry until further theoretical studies confirm our ability to infer more distant ancestries. The approaches developed here could be extended to allow more generations of hybridization but computational expense will increase dramatically.

Finally, though Mongrail does not require any fixed differences (or, exclusive alleles) between the 2 populations, high levels of genetic differentiation increase the power of the method to identify hybrids. In particularly, for the empirical data analysis, we found the SO genomes had little polymorphism and were very distinct from the much more variable BO genomes. The exceptionally low genetic diversity of SO genomes is likely due to a recent population decline. This may explain the high posterior probabilities we obtained as resulting from strong genetic differentiation between the 2 species.

Mongrail currently requires knowledge of haplotype frequencies in source populations. If this information is unavailable we need “pure” individuals (no recent hybrid ancestry) to be present in the sample in order to estimate these parameters. This implies that the 2 pure populations should be clearly distinguishable. One can be fairly confident of choosing individuals who are likely to be pure if sampled from 2 nonoverlapping geographical regions. This condition is automatically ensured for most allopatric populations. It is currently difficult to apply Mongrail to individuals sampled from a sympatric region when the 2 species (populations) cannot be separated or to a population with clinal variation of haplotype frequencies. To address this issue, a possible extension of our approach could be to jointly infer population haplotype frequencies and genealogical classes. This would require developing a MCMC method similar to the one presented in Anderson and Thompson (2002) (which jointly infers allele frequencies and genotype frequency classes). If pure individuals can be distinguished, it is recommended that at least 10 individuals are sampled from each population to produce sensible estimates of population haplotype frequencies. An important question, beyond the scope of the current study, concerns the effect of population sample size on errors of estimates of population haplotype frequencies (and resulting genealogical classifications).

Analyzing larger number of markers per chromosome has little effect in increasing the power to infer genealogical classes, yet an increased number of markers requires more haplotype frequencies to be estimated. Thus, there appears to be a tradeoff between information gained from additional markers and cost incurred by additional parameter estimates.

In conclusion, the method presented in this paper has the power to infer hybrids using linked genetic data without the requirement for any fixed or exclusive alleles to be present between 2 diploid populations. Extensive simulations show the potentially adverse effects of applying the widely used program NewHybrids (which assumes unlinked loci) to genomic data composed of large numbers of linked markers. Anderson and Thompson (2002) advised against using NewHybrids on tightly linked loci as it “will cause one to overestimate one’s certainty in identifying species hybrids.” The fact that the number of chromosomes, and the size of the intervals, contribute more to power than the number of markers allows an exact likelihood approach to be developed that is powerful without an excessive computational burden. Due to the analytical nature of the theory and consequent absence of simulation-based methodologies (such as MCMC) from the inference procedure the method is computationally efficient so that most of the runs finish within a few minutes. Rapid advances in sequencing technologies and bioinformatics tools, along with decreasing costs of genome sequencing and assembly, will increase the availability of genomic datasets for hybridizing nonmodel organisms. Therefore efficient statistical methods for identifying hybrids, such as Mongrail, that account for linkage will be increasingly needed in conservation biology and related disciplines.

## Supplementary Material

iyad011_Supplementary_Data

## Data Availability

Simulated datasets and scripts for generating simulations are available at https://github.com/mongrail/simulations. Scripts for analyzing the empirical dataset are available at https://github.com/mongrail/scripts. The Open Source C program Mongrail, implementing the algorithms presented in this paper, is available at https://github.com/mongrail. The owl dataset analyzed in this paper is publicly available at https://trace.ncbi.nlm.nih.gov/Traces/sra/sra.cgi?analysis=SRZ190173. Supplemental material available at *GENETICS* online.
