# Supplementary material for: An efficient exact algorithm for identifying hybrids using population genomic sequences
Source: Genetics. 2023 Jan 27;223(4):iyad011. doi: 10.1093/genetics/iyad011 (PMC10078916; doi:10.1093/genetics/iyad011)
Supplement: iyad011_Supplementary_Data [file iyad011_supplementary_data.pdf]

Supplementary Material for An Efficient Exact Algorithm for Identifying Hybrids Using Population Genomic Sequences

1 Simulation Study

Here, we describe the details of the simulation procedures used in the paper and of the statistical methods used to summarize the results in making a comparison of the statistical performance of Mongrail and NewHybrids.

1.1 Simulation methods

The simulation experiment used a factorial design allowing the performance of the methods to be assessed for many combinations of parameters. The parameters (factors) and their values were as follows:

1. Number of chromosomes: $K = 1, 2, 5, 10, 20$
2. Number of loci per chromosome: $L = 1, 5, 10$
3. Expected recombination frequency (in cM): $R = 1, 25, 50$ between the first and the last locus
4. Number of distinct haplotype sequences per chromosome for each population: $h = 5, 10, 15$
5. Allelic configurations of haplotypes, generated by simulating the switches between allele states (see Section 1.1.1). Switch rates used: $c = 0.1, 1, L/2$
6. Haplotype frequencies, following a Dirichlet distribution (symmetrical with parameters $\alpha = 1, 5$, or non-symmetrical with parameters $w = 5, 20$) (see Section 1.1.2)

For simplicity, in all simulations we fixed the length of each chromosome (D) to be 240 Mb and the recombination rate (r) to be 1.2 cM/Mb respectively.

The R scripts for simulating haplotype configurations (Section 1.1.1), simulating haplotype frequencies (Section 1.1.2) and simulating marker positions (Section 1.1.3) are available at Scripts.

1.1.1 Simulating haplotype configurations

The parameters for the simulation study were generated using R version 3.6.3. To simulate haplotype configurations we mimic recombination by using a "switching process" (that flips the adjacent marker state) operating along the chromosome. The switch rate on a particular interval is $p = c/L$ (when $L \neq 1$) where p is the probability of a switch from 0 to 1 (or 1 to 0).
The allele state for the first marker is simulated randomly from a Bernoulli(1/2) distribution. Given the value of L and c, the allele states for the rest of the $(L-1)$ markers are simulated from a Bernoulli(p) distribution (following the description in the previous paragraph). We repeat the process above until we obtain h distinct haplotypes. These simulations were performed using the *rbinom* function in R.

When $L = 1$, there are only two possible haplotypes 0 and 1 (the two allele states possible for a single marker). Thus for $L = 1$, the number of haplotypes $h = 2$.

We use the same haplotype configurations for both the populations (A and B) for each of the K chromosomes.

1.1.2 Simulating haplotype frequencies

Given the h distinct haplotypes generated in section 1.1.1, we simulate their corresponding population frequencies. Let $\mathbf{f_k^A}$ and $\mathbf{f_k^B}$ be the haplotype frequencies in population A and B respectively for chromosome k for $k = 1, 2, \ldots, K$. We perform these simulations using either a symmetrical or non-symmetrical Dirichlet distribution. The simulations were generated using the *rdirichlet* function in R. We simulate h frequencies from a Dirichlet Distribution with parameters $\alpha_1, \alpha_2, \ldots, \alpha_h$.

For the symmetric Dirichlet distribution we set $\alpha_i = \alpha$ for $i = 1, 2, \ldots, h$ and consider two cases: $\alpha = 1$ or $\alpha = 5$.

For the non-symmetric Dirichlet distribution we use:

$$\alpha_i = \begin{cases} 0.7 \times w, & i = 1 \\ 0.3 \times \frac{w}{h-1}, & i > 1, \end{cases}$$

and again consider two cases: $w = 5$ or $w = 20$.

When $L = 1$, there are only 2 haplotypes, and we simulate $h = 2$ frequencies from Beta distributions (univariate cases of the Dirichlet distribution) with parameters α_1 and α_2:

1. $\alpha_1 = \alpha_2 = 1$
2. $\alpha_1 = \alpha_2 = 5$
3. $\alpha_1 = 0.7 \times 5, \alpha_2 = 0.3 \times 5$
4. $\alpha_1 = 0.7 \times 20, \alpha_2 = 0.3 \times 20$

We repeat this process for each of the K chromosomes.

1.1.3 Assigning marker positions

Given the value of $L(\neq 1), R$ and r, we calculate the distance between the first and last marker in units of base pair (bp) which is

$$\frac{R \times (10^6)}{r}.$$

We consider all L markers to be equidistant from each other. Therefore the inter-marker distance (in units of bp) is given by

$$\frac{R \times (10^6)}{r \times (L-1)}.$$

For simplicity, we consider the position of the first marker (in bp) as $\frac{R \times (10^6)}{r}$ and obtain the positions of the rest of the $L - 1$ markers (in bp) following the description above. For $L = 1$, we arbitrarily chose 120000bp as the marker position. We use the same marker positions for both the populations (A and B) for each of the K chromosomes.

1.2 Simulating diplotypes for markers

All simulations in this section were done in the C programming environment. This simulation environment is available as an option in our program Mongrail. Given that the haplotypes and their corresponding frequencies (along with the marker positions) have been generated for the two populations A and B, generating a diploid individual is equivalent to generating a diplotype (a pair of haplotypes). Generating a diplotype from any of the models: **a** (pure population B), **c** (F1 hybrid) or **d** (pure population A) is straightforward. For these models, each of the two chromosomes derives entirely from one population (A or B). To simulate a chromosome, a sample of size 1 from a multinomial distribution with h types was simulated, where h is the number of haplotypes, and the multinomial proportions $\mathbf{f^j}$ are the haplotype frequencies in the source population, $j \in \{\text{A}, \text{B}\}$. For this purpose we

used the function *gsl_ran_multinomial* from the GNU scientific library. For model **a** (or, **d**) both the chromosomes are simulated independently from population B (or, A) respectively. In case of model **c**, one chromosome is simulated from population A and the other from population B.

For the other models, one (**b**, **e**) or both (**f**) the chromosomes are recombinant (a mixture between two pure parental chromosomes, one from population A and the other from B). Recombinant chromosomes were simulated in two steps:

Step I

Simulate two pure parental chromosomes (one from population A and the other from B) which is equivalent to simulating from model **c** (described above). Denote the two simulated pure chromosomes as C_A (Population A) and C_B(Population B) respectively. For example, let's say we generated the following two chromosomes for $L = 10$ markers:

$$C_A \equiv 1100011010$$

$$C_B \equiv 0111001001$$

Here the 0 or 1 indicate the allele present at each marker since we consider phased biallelic SNP markers. Therefore C_A (or, C_B) is a binary string of size L. We shall carry on with this example in the following steps.

Step II

Simulate recombinations between the two pure chromosomes to produce a pair of recombinant chromosomes. This is achieved as follows:

(a) **Simulate the number of recombinations:** We simulate the number of recombinations (n_r) over the length of the chromosome. Assuming the rate of recombination over the chromosome is uniform, and recombination events are independent and never occur simultaneously, n_r follows a Poisson distribution with rate parameter λ (the expected number of recombination events over the entire chromosome). Given the rate of recombination (r) in units of cM/Mb and the length of the chromosome (say, D) in units of Mb, the map distance of the chromosome in units of centiMorgans (cM) is $D \times r$ and $\lambda = (D \times r)/100$. We used the function *gsl_ran_poisson* from GNU Scientific Library to generate n_r.

(b) **Simulate the positions of recombinations:** The positions of recombinations on the chromosome, conditional on the number of recombinations (n_r), are simulated from a continuous uniform distribution over the length of the chromosome (in cM). For example, if $n_r = 3$, we might generate a single crossover event between each of the following pair of marker positions: $(3, 4)$, $(5, 6)$ and $(7, 8)$. We used the function *gsl_rng_uniform_pos* from GNU Scientific Library to generate the recombination positions.

(c) **Obtain the population origin of markers:** The population origin (or, ancestry state) of the markers was obtained conditional on the positions of recombinations. The population origin of a SNP marker to the right of an interval changes whenever there is an odd number of recombinations (similarly, an even number of recombinations results in no change). We move from left i.e., 5' end of the chromosome to the right towards the 3' end of the chromosome. Based on the example developed so far, two haplotype ancestry states are produced for $L = 10$ markers:

$$AAABBAABBB \quad \text{and} \quad BBBAABBAAA$$

(d) **Obtain the allelic state (0/1) of the markers:** The allele type (0 or 1) for each marker is derived from C_A or C_B depending on whether the ancestry state is A or B respectively for the marker under consideration. For the particular C_A and C_B we considered at the beginning and based on the ancestry state $AAABBAABBB$, the alleles at the markers from position 1 to 3 and from positions 6 to 7 should come from population A. This requires that the alleles at

these positions should match those of C_A (indicated by the underbraces) and the alleles for the rest of the markers match those of chromosome C_B.:

$$C_A \equiv \underbrace{110}\,00\,\underbrace{11}\,010$$

$$C_B \equiv 011\,\underbrace{10}\,01\,\underbrace{001}$$

Therefore, given the ancestry state $AAABBAABBB$, the allele states of the markers for one recombinant haplotype are

$$r_{H_1} \equiv 1101011001.$$

Similarly, given the ancestry state is $BBBAABBAAA$, the allele states of the markers for the other haplotype are

$$r_{H_2} \equiv 0110001010.$$

Thus, there are two r_{H_1} and r_{H_2} recombinant haplotypes. We used bit operations in C to perform this step.

(e) **Simulate a recombinant chromosome:** The two recombinant chromosomes (r_{H_1} and r_{H_2}) are equally likely to occur. So we choose one of them from a Bernoulli(1/2) distribution (say, getting r_{H_1} is defined as success). We used the function *gsl_ran_bernoulli* from GNU Scientific Library to generate a recombinant chromosome.

1.3 Coalescent Simulation

1.3.1 Simulating diplotypes

Here we describe the procedure for generating diplotypes under different genealogical classes given a sample of chromosomes from two populations simulated under the structured coalescent model. Generating a diplotype which is a purebred (model **d**) or F1 (model **c**) is straightforward. Under model **d** both the chromosomes arise entirely from population B. To simulate a purebred diploid individual from population B, we simulate 2 additional chromosomes from population B in the coalescent simulation; the two chromosomes from the purebred diplotype. In an F1 hybrid, one chromosome arises from population A and the other from population B. To simulate an F1 hybrid, we simulate 1 additional chromosome from each of the two populations (A and B) during the coalescent simulation, the two chromosomes form the F1 diplotype. For a backcross (model **b**) one chromosome arises entirely from population A and the other is a recombinant between a pair of chromosomes, one from population A and the other from B. Therefore we simulate 3 additional chromosomes (2 from population A and 1 from population B) and follow Step II of Supplementary Data Section 1.2 to produce a recombinant chromosome (using one of the chromosomes from population A and another from B). The A-B recombinant and A chromosomes together form the backcross diplotype. For an F2 hybrid (model **f**) both chromosomes are recombinant between populations A and B. To simulate an F2 hybrid 4 additional chromosomes are simulated (2 from population A and 2 from population B) during the coalescent simulation and each pair (one chromosome from A and one from B) undergo recombination according to Step II of Supplementary Data Section 1.2. The two A-B recombinant chromosomes together form the F2 diplotype.

1.3.2 Summary of number of population haplotypes generated

We computed the mean and standard deviation of the number of distinct haplotypes observed in populations A and B under the coalescent simulation. These results are presented for all values of $M = 0.1, 0.25, 1, 10, 100$ in Table S1. As the value of M increases, the mean number of unique haplotypes increases in both simulated populations. This is expected as an increase in migration introduces additional shared haplotypes into both populations. Since the coalescent simulation was carried out under a symmetric island model the two populations are equivalent and thus the mean and standard deviation are nearly identical between the two populations.

Table S1: The mean and standard deviation (S.D.) of the number of distinct 10-locus haplotypes generated under each coalescent simulation scenario for two populations A and B.

Migration Rate (M)	Population A		Population B	
	Mean	S.D.	Mean	S.D.
0.1	9.17277	5.7704	9.1582	5.74928
0.25	13.3307	7.23048	13.3239	7.25902
1	17.9721	8.62542	17.9387	8.62992
10	21.3009	8.84554	21.2143	8.78405
100	22.6983	8.89778	22.6209	8.85955

1.4 Evaluating statistical performance

1.4.1 Accuracy of posterior probabilities

The aim of this analysis was to verify that the posterior probabilities of genealogical classes for individuals provide consistent and unbiased estimates of the frequencies at which individuals belong to the classes. The underlying true genealogical class is known for a simulated individual and a Bayesian method should produce posterior probabilities for genealogical classes (models) that correspond to the frequency at which that genealogical class is the one under which the individual was simulated. To evaluate this, individual posterior probabilities for the gth genealogical class were binned into 10 intervals each of length 0.1. The proportion of individuals in each bin for which the true genealogical class was g was calculated and plotted against the mid-point of the posterior probability for the interval. The method is performing well if, in each interval with midpoint posterior probability p, the frequency of individuals binned into that interval for whom the true genealogical class is g is close to p. This will produce a straight line along the diagonal of the plot. This expected relationship holds for all the six genealogical classes. For example, 95% of individuals that are placed in bin $(0.9, 1.0)$ for genealogical class g should have true genealogical class g while the remaining 5% will have a true genealogical class that belongs to one of the other five alternatives.

1.4.2 Power to identify genealogical classes

The aim of this analysis was to examine how often a method produces a high posterior probability for the true genealogical class used to simulate an individual. If the posterior probability is accurate, a greater proportion of high posterior probability outcomes will indicate greater power. The posterior probability assigned to an individual for each of the six genealogical classes was plotted as a stacked bar plot (Figure 4). Different colors are used to illustrate the different segments in the bar. Each colored segment represents the relative contribution of one of the six genealogical class posterior probabilities for that individual. One can conclude the method is performing well for individuals simulated under the gth genealogical class if the color assigned to the gth class is the dominant one in the plot. If the bar has many colors with uniform representation there is low support for any particular model and thus low power.

1.4.3 Posterior distribution of genealogical classes

The aim of this analysis was to examine the posterior probability of the true genealogical class for individuals simulated under that genealogical class. If a method is powerful most individuals should have a posterior probability concentrated near posterior probability 1 for the true genealogical class (a high frequency of individuals at the right of the graph). If a method is accurate few individuals should have a posterior probability concentrated near posterior probability 0 for the true genealogical class (a low frequency of individuals at the left of the graph).

1.4.4 ROC curve analysis of power versus Type I error

The aim of this analysis was to examine the power of the methods to detect genealogical classes relative to Type I error for different classification thresholds. We plot the ROC (Receiver Operating Characteristics) curve for all six genealogical classes. The ROC curve for the gth genealogical class is created by plotting the true positive rate (equivalent to power, or sensitivity) against the false positive rate (equivalent to Type I error, or $1-$ specificity). The true positive rate is defined as the proportion of individuals simulated under the gth genealogical class and classified as belonging to that class. The false positive rate is defined as the proportion of individuals simulated under another class but classified as belonging to the gth class. The ROC curve measures the performance of the methods for classifying individuals into genealogical classes as a function of varied classification thresholds (based on posterior probabilities). For each of the six genealogical classes we overlay the ROC curves of Mongrail and NewHybrids to allow comparisons between the two methods.

1.4.5 Sensitivity to biological and experimental parameters

The aim of this analysis was to determine how sensitive the method is to changes of key biological and experimental parameters: number of chromosomes (K), number of loci (L), and recombination frequency (R). All of these factors are affected both by the biology of the organism under study and by the experimental design. We expect increased information with increasing values of K, L or R. However, we do not know apriori how large the effect will be on the posterior probabilities of genealogical classes. To reduce the state space for this analysis, we consider fixed values for other parameters that determine haplotype frequencies in populations – these other paranmeters are typically beyond the experimentalists control and are also expected to have less predictable effects on method performance. We used: $h = 5, c = 0.1$ and $\alpha = 1$. We plot the proportion of cases for which the posterior probability of belonging to the correct genealogical class is above a threshold value of 0.9 against the number of chromosomes analyzed using multi-line plots, where each line represents different combinations of values for the remaining two parameters: number of loci (except $L \neq 1$) and recombination frequency (R).

1.5 Distribution of Linkage Disequilibrium (LD)

We obtained the linkage disequilibrium coefficient (r^2) under a particular set of simulation combinations (cases where haplotype frequencies were simulated using symmetric Dirichlet Distribution). We only consider the linkage disequilibrium coefficient (r^2) between the first and last marker. For brevity we plot the distribution of r^2 values for four specific simulation combinations (Figure S1):

1. $L = 5, h = 5, \alpha = 5, c = 1$
2. $L = 10, h = 15, \alpha = 1, c = 0.1$
3. $L = 5, h = 15, \alpha = 5, c = L/2$
4. $L = 5, h = 15, \alpha = 5, c = 0.1$

We find that under our comprehensive simulation setup, the r^2 values range from really low values (close to 0) to very high values (close to 1). Therefore this shows that the comprehensive simulation design does not explicitly produce high LD values. It produces a broad range of LD values ranging from 0 to 1.

2 Empirical Dataset

We apply our method to the set of the 51 owl samples shown in Table S2. We used the filtered Variant Call Format (VCF) file available at SRZ190173. The dataset includes 17,385,299 biallelic single nucleotide polymorphisms (SNPs) across 82 large autosomal scaffolds and 8,543,351 of these

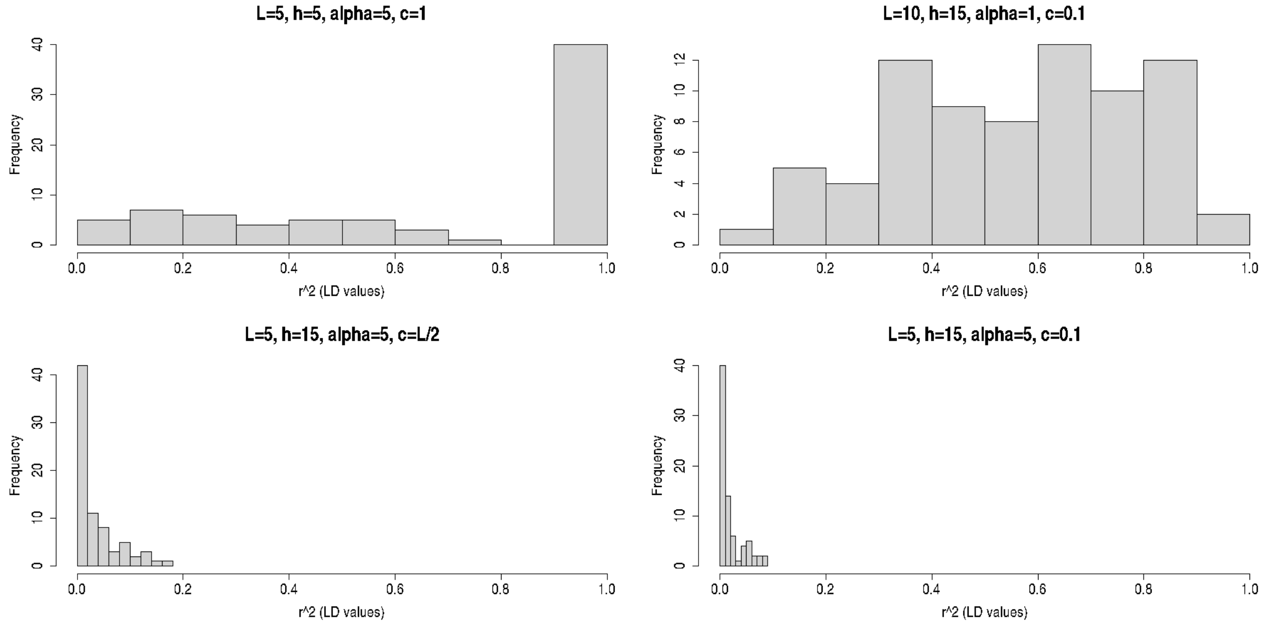

Figure S1: Histogram showing the frequency distribution of linkage disequilibrium coefficient (r^2) under four specific simulation combinations: (a) $L = 5, h = 5, \alpha = 5, c = 1$ (top-left), (b) $L = 10, h = 15, \alpha = 1, c = 0.1$ (top-right), (c) $L = 5, h = 15, \alpha = 5, c = L/2$ (bottom-left), (d) $L = 5, h = 15, \alpha = 5, c = 0.1$ (bottom-right).

had high-confidence genotype calls ($GQ \geq 40$) in all individuals. The sequence data were analyzed using BCFtools version 1.10.2 and BEAGLE version 5.1. Bash scripts used to perform the analyses are available at Scripts.

2.1 Data preprocessing and parameter estimation

We restricted our analyses to the 15 largest autosomal scaffolds and filtered out sites with missing genotypes for any individual. For each scaffold, we extracted the Spotted Owl (10 individuals, excluding Sequoia) and Barred Owl (25 individuals) populations into two separate VCF files and phased each population separately for each scaffold. The haplotype frequencies for the two populations were estimated using the Multinomial-Dirichlet posterior mean (see equation 1).

2.2 Analysis of putative hybrids

Marker genotypes were filtered for each scaffold using positions chosen such that they satisfied physical distances specified for the different analyses (either a sliding window analysis or a maximally informative distribution) using BCFtools, and reformatted for input to Mongrail using Awk and Sed. A C program was used to enumerate all compatible diplotypes for putative hybrid individuals for each choice of markers, which formed the input for Mongrail. For each scaffold and genealogical class, the total likelihood is obtained as a sum of the likelihood calculated for each compatible diplotype. When multiple scaffolds are analyzed, likelihoods are multiplied across scaffolds, treating scaffolds as equivalent to independent chromosomes. Posterior probabilities are obtained from the normalized likelihoods (using a uniform prior on genealogical classes). Results were plotted in R using Tidyverse, RColorBrewer and reshape.

2.3 Spatial variation of model posterior probabilities

The purpose of this analysis is to study the variation in posterior probabilities of genealogical classes for owl samples across the genome by using different sets of $L = 10$ loci from a particular scaffold region of variable map size ($R = 1.5$cM and $R = 50$cM). This was implemented using a sliding

Table S2: Table showing the sampling locations (states in North America) of the 51 owl samples consisting of 11 spotted owls, 25 barred owls, 2 known hybrids and 13 potential hybrids. The purebred samples (SO and BO) are further categorized into their recognized sub-species.

Species Category	State	#individuals	Total #individuals
Northern Spotted Owl	California	5	8
	Oregon	2	
	Washington	1	
California Spotted Owl	California	3	3
Eastern Barred Owl	Kentucky	2	12
	Ohio	2	
	New York	3	
	Massachusetts	3	
	New Jersey	1	
	Indiana	1	
Western Barred Owl	California	13	13
Putative Hybrid	California	7	13
	Oregon	5	
	Washington	1	
Known Hybrid	California	1	2
	Oregon	1	

window approach over an entire scaffold starting from the 5' end and moving towards 3'. A window implies a set of L markers each of length R, where the markers are almost equidistant to each other. We say "almost" since the theoretically equidistant marker positions are not all present in the actual dataset. The recombination rates for SO and BO are not available, so following Hanna et al. (2018) we assumed a recombination rate of 1.5 cM/Mb (the average recombination rate of zebra finch). At $R = 50$cM (a physical length of 33.33 Mb), we chose 100 initiating markers to create windows evenly spaced across the scaffold. Then for every window the posterior probability assigned to an owl sample for each of the six genealogical classes was plotted as a stacked bar plot. Different colors are used to illustrate the different segments in the bar. Each colored segment represents the relative contribution of one of the six genealogical class posterior probabilities for that owl sample. Using the same 100 initiating markers but with $R = 1.5$cM (a physical length of 1 Mb) we created similar stacked bar plots. The two stacked bar plots are compared to examine the effects of varying map length on posterior probabilities. Within each plot we examine the consistency of the posterior probabilities across the entire scaffold.

2.4 Effect of successive scaffold inclusion for varied map length

The purpose of this analysis is to examine the variation in posterior probabilities of genealogical classes for owl samples as we increase the number of scaffolds for different levels of recombination frequency. For each sample we considered $L = 10$ markers from all 15 scaffolds where the scaffolds are arranged in a decreasing order of length. We study the cumulative effect of adding the scaffolds successively starting from the largest scaffold (Super-Scaffold_7) of size 72.11 Mb to the smallest scaffold (Super-Scaffold_47) of size 21.02 Mb on the posterior probabilities. A recombination rate of 1.5 cM/Mb is assumed for the same reasons as mentioned earlier. We perform the analysis under two cases. In the first case, we use a constant recombination frequency (or, map length) of $R = 1.5$cM (a physical length of 1Mb) for every scaffold. For the second one, we vary the recombination frequency over each scaffold in a way such that its equivalent physical length is approximately equal (rounded off to a whole number) to the length of the scaffold. Say, for the largest scaffold which is of length 72.11 Mb we use a map length of 108cM which is equivalent to a physical length of 72 Mb. This design that incorporates almost the entire scaffold size as the map length has the advantage of retaining maximum information. Henceforth we refer to this second case as "maximally informative". Now we need to choose a set of $L = 10$ markers from each scaffold. Here we adopt the

window approach (as described in the previous section) for each scaffold and arbitrarily select the middlemost window under this "maximally informative" case. For every owl sample we plot the posterior probability for the gth genealogical class against the cumulative number of scaffolds using multi-line plots, where each line represents a particular genealogical class. From the simulation analysis we found that increasing the number of chromosomes results in an increase in information. So we examine this multi-line plot to see whether a particular genealogical class is favored over others as the number of scaffolds increases. Ideally the gth genealogical class is said to be "preferred" over the rest, if the posterior probability for the gth genealogical class approaches one while the rest of the lines concentrates around zero as the number of scaffold increases. Using the same initiating markers as chosen earlier for each scafffold but with a constant map length of $R = 1.5$cM we produce similar multi-line plots. The multi-line plots for the two cases of recombination frequency are compared to study the effects of different map length on posterior probabilities.

2.5 Sensitivity of results to assumed recombination rate

In the previous two analyses we studied the behaviour of the posterior probabilities of genealogical classes under a fixed recombination rate of 1.5 cM/Mb. This value has been extrapolated from another species, the zebra finch (*Taeniopygia guttata*) as the recombination rate for owls was not available. This raises the question whether the choice of recombination rate affects the inference of genealogical classes. We conducted a sensitivity analysis to study the behaviour of the posterior probabilities of the "preferred" model (inferred in the previous analyses) for different values of the recombination rate when successively increasing the number of scaffolds. We chose three different levels of recombination rate (in cM/Mb): $r = 0.5, 1.5$ and 5. For each individual we plot the posterior probability of the "preferred" genealogical class against the cumulative number of scaffolds using multi-line plots, where each plot represents a particular value of recombination rate r. The map length used for scaffolds is similar to the "maximally informative" case described above.

2.6 Assignment of 15 hybrids (13 putative and 2 known)

This analysis aimed to compare the genealogical classifications obtained from Mongrail with previous classifications of the same individuals. We used all 15 scaffolds and assumed a recombination rate of 1.5cM/Mb (with a "maximally informative" region size) to construct a table of the posterior probabilities of the "preferred" model for each of the 15 hybrids (along with the primary and genetic identification information of Fujito et al., 2021). The purpose of this table was to examine whether posterior probabilities of the "preferred" model are high enough to make an assignment call for each of the 15 hybrid owl samples using a pre-specified threshold posterior probability (for example, 0.99). Given that an individual can be classified, we examined whether our inference matched the previous genetic identification (Fujito et al., 2021).

3 Applying NewHybrids to the owl dataset

3.1 Assignment of 15 hybrids (13 putative and 2 known)

We applied NewHybrids to the owl dataset to obtain genealogical classifications of the putative hybrids. We used the same set of 10 markers from the 15 scaffolds that were considered for Mongrail (the case where the assumed recombination rate was 1.5cM/Mb under a "maximally informative" region size). Therefore we used 150 markers in total to construct a table (see Table S3) of the posterior probabilities of the "preferred" model for each of the 15 hybrids (along with the primary and genetic identification information of Fujito et al., 2021).
Based on the NewHybrids model framework, genealogical class d, c and b refers to a pure barred owl, a F1 hybrid and a backcross with barred owl respectively. The results are similar to those obtained by applying Mongrail to this owl dataset (see Table 2). The preferred model has posterior probability 1 for all samples suggesting that both Mongrail and NewHybrids similarly provide reasonable results that align with previous conclusions.

Sample Names	Primary Identification	Genetic Identification	Posterior Probability	Preferred Model
TLW521	Unknown	Barred Owl	1	d
TLW532	Unknown	Barred Owl	1	d
AFRD90	Unknown	Barred Owl	1	d
CYWC009	Unknown	Barred Owl	1	d
1957-00137	Unknown	F1	1	c
1957-00240	Unknown	F1	1	c
1957-00243	Unknown	F1	1	c
LCW1363	Unknown	F1	1	c
LCW1383	Unknown	F1	1	c
ZRH600	Unknown	F1	1	c
ZRH610	Unknown	F1	1	c
ZRH962	Known Hybrid	F1	1	c
TLW519	Unknown	Backcross	0.99912	b
TLW528	Unknown	Backcross	1	b
ZRH607	Known Hybrid	Backcross	1	b

Table S3: Table showing the assignment call for all 15 hybrid owl samples using NewHybrids.

3.2 Comparison of posterior probability distributions

In this analysis we compare the posterior probabilities under the two methods (Mongrail and NewHybrids) for 5 putative hybrids. These owls were chosen to be representative of the three observed categories genetically identified by Fujito et al. (2021). The categories along with the sample names are presented in Table S4.

Primary identification	Genetic identification	Sample names
putative hybrid	BO	CYWC009
known hybrid	F1 hybrid (F1)	ZRH962
putative hybrid	backcross (F1× BO)	TLW519
putative hybrid	backcross (F1× BO)	TLW528
known hybrid	backcross (F1× BO)	ZRH607

Table S4: Details for 5 individuals chosen for detailed analysis. Primary and genetic identifications are from Fujito et al. (2021).

We chose 10 markers from the largest scaffold (Super-Scaffold_7 of length 72.11 Mb) and consider two different map lengths. We considered $R = 1.5$ cM and the "maximally informative" case described in section 2.4 .
There is a greater distribution of posterior probabilities among genealogical classes under Mongrail by comparison with NewHybrids (compare top and bottom rows in Figures S2 and S3). Even with 10 markers, NewHybrids tends to produce extremely high posterior probabilities for specific genealogical classes for both small (Figure S3) and large (Figure S2) intervals. Thus NewHybrids seems to be overconfident even when information is quite low. In one case (individual TLW519) the posterior probability for genealogical class d is nearly one in the "maximally informative" case (Figure S2), whereas for a smaller interval ($R = 1.5$ cM) the posterior probability for genealogical class d drops to 0.02 and the posterior probability for genealogical class c increases to 0.83 (Figure S3). In both cases the genealogical class with the highest posterior probability differs from the preferred genealogical class. Mongrail appears more conservative, never assigning a posterior probability to a non-preferred model of more than 0.30 for individual TLW519.

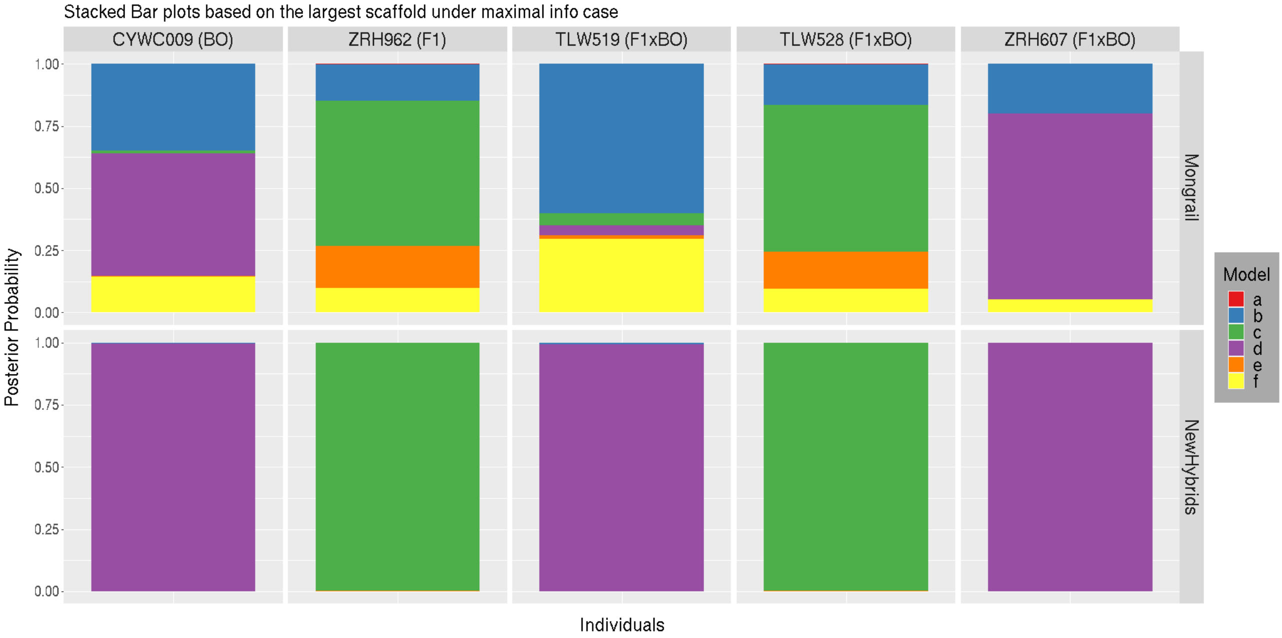

Figure S2: Distribution of posterior probabilities is constructed for 5 owl samples (CYWC009, ZRH962, TLW519, TLW528, ZRH607). A set of $L = 10$ markers are chosen from the largest scaffold Super-Scaffold_7 (size 72.11 Mb) according to the “maximally informative” case. For each individual, posterior probabilities for Mongrail are shown in the top plot and for NewHybrids in the bottom plot. The posterior probabilities for different genealogical classes are represented by segments of different colors. The proportion of the stacked bar plot comprised of a particular color indicates the posterior probability of the model corresponding to that color. The 6 genealogical classes are as follows: **a**-pure population B, **b**-backcross with population A, **c**-F1 hybrid, **d**-pure population A, **e**-backcross with population B, **f**-F2 hybrid. In our model framework, BO is treated as population A and SO as population B.

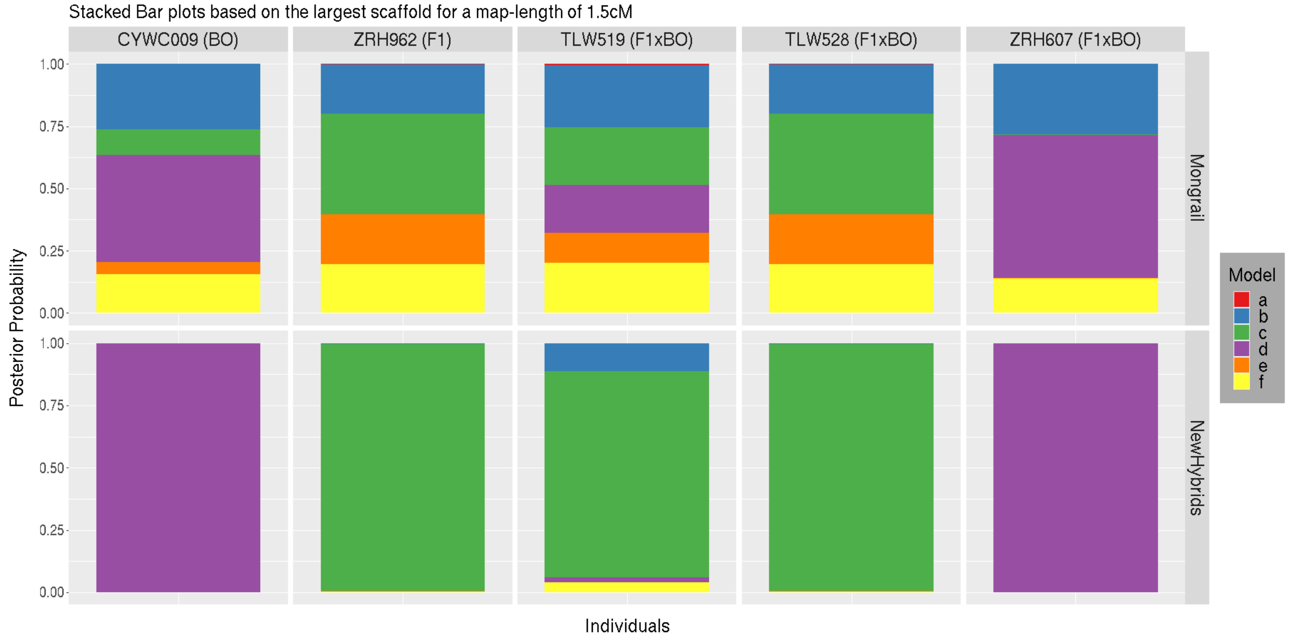

Figure S3: Distribution of posterior probabilities is constructed for 5 owl samples (CYWC009, ZRH962, TLW519, TLW528, ZRH607) for a set of $L = 10$ markers, where the markers are evenly spaced across the largest scaffold Super-Scaffold_7 (size 72.11 Mb) with a constant map length of $R = 1.5$cM. The initiating marker is same as the first marker in the "maximally informative" case. For each individual, posterior probabilities for Mongrail are shown in the top plot and for NewHybrids in the bottom plot. The posterior probabilities for different genealogical classes are represented by segments of different colors. The proportion of the stacked bar plot comprised of a particular color indicates the posterior probability of the model corresponding to that color. The 6 genealogical classes are as follows: **a**-pure population B, **b**-backcross with population A, **c**-F1 hybrid, **d**-pure population A, **e**-backcross with population B, **f**-F2 hybrid. In our model framework, BO is treated as population A and SO as population B.

3.3 NewHybrids analysis using unlinked SNPs with fixed differences

To explore the effects of fixed differences between these two species on hybridization inference with NewHybrids, we applied NewHybrids to the same set of 5 putative hybrids analyzed previously (See Table S4) but specifically chose loci with fixed differences. We randomly chose a single marker from among all the markers with fixed differences for each of the 15 scaffolds. As shown in Figure S4, when loci are fixed for alternate alleles NewHybrids produces extremely high posterior probabilities (greater than 0.998) for specific genealogical classes, even when using only 15 markers. For the individuals analyzed, the "preferred" genealogical class matches the prior genetic classification. Caution is needed when considering such results, since without exhaustive population sampling it is always uncertain whether "fixed" differences are truly fixed or instead an artifact of genotyping error or failure to sample an allele.

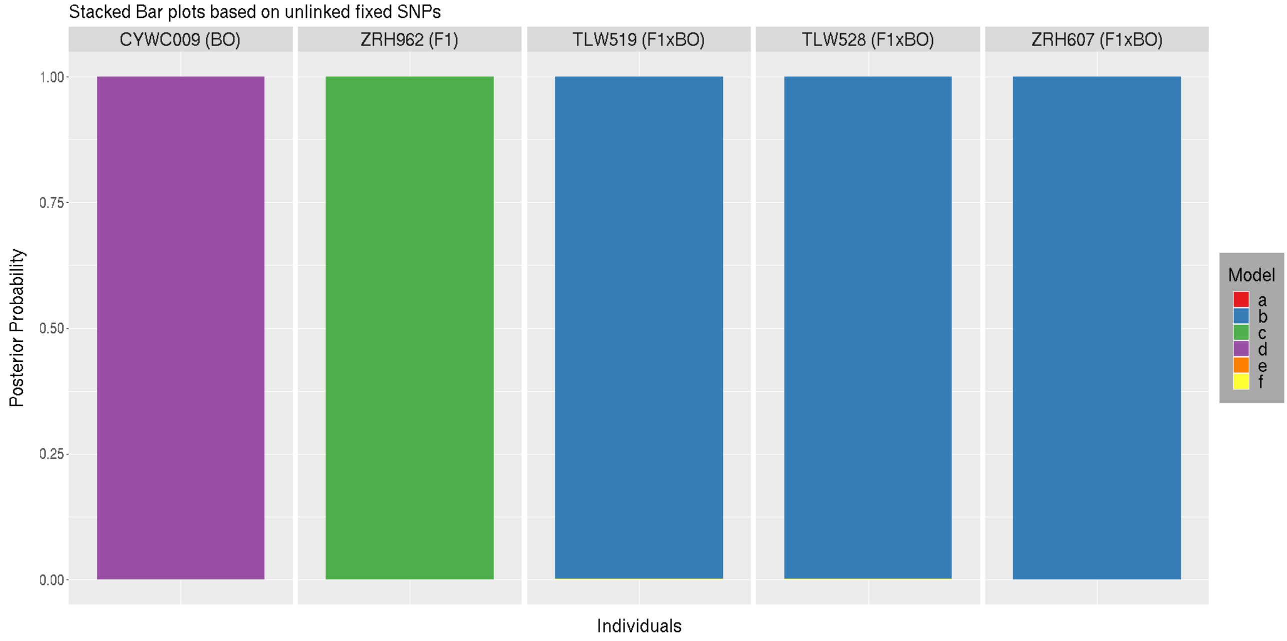

Figure S4: Distribution of posterior probabilities for 5 owl samples (CYWC009, ZRH962, TLW519, TLW528, ZRH607) obtained by applying NewHybrids to a set of 15 fixed SNPs, where each SNP is chosen randomly from each of the 15 scaffolds. The posterior probabilities for different genealogical classes are represented by segments of different colors. The proportion of the stacked bar plot comprised of a particular color indicates the posterior probability of the model corresponding to that color. The 6 genealogical classes are as follows: **a**-pure population B, **b**-backcross with population A, **c**-F1 hybrid, **d**-pure population A, **e**-backcross with population B, **f**-F2 hybrid. In our model framework, BO is treated as population A and SO as population B.

References

Fujito, N. T., Z. R. Hanna, M. Levy-Sakin, R. C. Bowie, P.-Y. Kwok, et al., 2021 Genomic variation and recent population histories of spotted (strix occidentalis) and barred (strix varia) owls. Genome Biology and Evolution **13**: evab066.

Hanna, Z. R., J. P. Dumbacher, R. C. Bowie, J. B. Henderson, and J. D. Wall, 2018 Whole-genome analysis of introgression between the spotted owl and barred owl (strix occidentalis and strix varia, respectively; aves: Strigidae) in western north america. G3: Genes, Genomes, Genetics **8**: 3945–3952.
